# Comprehensive immunophenotyping of solid tumor-infiltrating immune cells reveals the expression characteristics of LAG-3 and its ligands

**DOI:** 10.3389/fimmu.2023.1151748

**Published:** 2023-09-19

**Authors:** Bradley Garman, Can Jiang, Sherif Daouti, Sanah Kumar, Priyanka Mehta, Miye K. Jacques, Laurence Menard, Nataly Manjarrez-Orduno, Sonia Dolfi, Piali Mukherjee, Sharmila Chamling Rai, Ana Lako, Jennifer D. Koenitzer, Justin M. David

**Affiliations:** ^1^ Translational Medicine, Bristol Myers Squibb, Lawrenceville, NJ, United States; ^2^ Epigenomics Core Facility, Weill Cornell Medicine, New York City, NY, United States

**Keywords:** Immunophenotyping, Immunotherapy, TIL, LAG-3, PD-1, CD8 memory T cell, MHC-II, galectin-3

## Abstract

**Background:**

Immune cell expression profiling from patient samples is critical for the successful development of immuno-oncology agents and is useful to understand mechanism-of-action, to identify exploratory biomarkers predictive of response, and to guide treatment selection and combination therapy strategies. LAG-3 is an inhibitory immune checkpoint that can suppress antitumor T-cell responses and targeting LAG-3, in combination with PD-1, is a rational approach to enhance antitumor immunity that has recently demonstrated clinical success. Here, we sought to identify human immune cell subsets that express LAG-3 and its ligands, to characterize the marker expression profile of these subsets, and to investigate the potential relationship between LAG-3 expressing subsets and clinical outcomes to immuno-oncology therapies.

**Methods:**

Comprehensive high-parameter immunophenotyping was performed using mass and flow cytometry of tumor-infiltrating lymphocytes (TILs) and peripheral blood mononuclear cells (PBMCs) from two independent cohorts of samples from patients with various solid tumor types. Profiling of circulating immune cells by single cell RNA-seq was conducted on samples from a clinical trial cohort of melanoma patients treated with immunotherapy.

**Results:**

LAG-3 was most highly expressed by subsets of tumor-infiltrating CD8 T central memory (TCM) and effector memory (TEM) cells and was frequently co-expressed with PD-1. We determined that these PD-1^+^ LAG-3^+^ CD8 memory T cells exhibited a unique marker profile, with greater expression of activation (CD69, HLA-DR), inhibitory (TIM-3, TIGIT, CTLA-4) and stimulatory (4-1BB, ICOS) markers compared to cells that expressed only PD-1 or LAG-3, or that were negative for both checkpoints. In contrast to tumors, LAG-3 expression was more limited in circulating immune cells from healthy donors and solid tumor patients. Additionally, we found abundant expression of the LAG-3 ligands MHC-II and galectin-3 in diverse immune cell types, whereas FGL1 and LSECtin were minimally expressed by immune cells in the tumor microenvironment (TME). Lastly, we found an inverse relationship between baseline and on-treatment levels of circulating LAG3 transcript-expressing CD8 memory T cells and response to combination PD-1 and CTLA-4 blockade in a clinical trial cohort of melanoma patients profiled by scRNAseq.

**Conclusions:**

These results provide insights into the nature of LAG-3- and ligand-expressing immune cells within the TME, and suggest a biological basis for informing mechanistic hypotheses, treatment selection strategies, and combination immunotherapy approaches to support continued development of dual PD-1 and LAG-3 blockade.

## 1 Introduction

The development of immuno-oncology (IO) agents in recent years has revolutionized the treatment of cancer. Current approved approaches include targeting T cell checkpoints, and these therapies, alone or in combination, have led to remarkable clinical benefits for many patients with previously untreatable disease. However, there remains a proportion of patients whose tumors do not respond or who relapse following an initial response to these immunotherapies, and therefore require the development of alternative treatment strategies (1).

One approach has been to combine programmed cell death 1 (PD-1) and/or cytotoxic T-lymphocyte associated protein 4 (CTLA-4) checkpoint inhibitors with the targeting of additional distinct immune pathways for novel IO-based combinations. In particular, additional T-cell checkpoint inhibitory receptors, including lymphocyte activation gene-3 (LAG-3), T cell immunoglobulin and mucin domain-3 (TIM-3), T cell immunoreceptor with immunoglobulin (Ig) and ITIM domains (TIGIT), B and T lymphocyte attenuator (BTLA), and V-domain immunoglobulin-containing suppressor of T-cell activation (VISTA) have been proposed as potential targets for therapeutic combination development (2). Among these, LAG-3 became the third clinically validated checkpoint target, with the recent RELATIVITY-047 (NCT03470922) data resulting in FDA approval of the anti-LAG-3 antibody relatlimab in combination with the anti-PD-1 antibody nivolumab for the treatment of unresectable or metastatic melanoma (3, 4). LAG-3 is an inhibitory immune receptor known to be expressed by activated CD4 and CD8 T cells, regulatory T cells (Tregs), natural killer (NK) cells, and plasmacytoid dendritic cells (pDCs) (5). It is a member of the Ig superfamily and exhibits structural homology to CD4. Like CD4, it binds to major histocompatibility complex (MHC)-II, but with a much higher affinity (6). In addition to MHC-II, other known ligands of LAG-3 include liver and lymph node sinusoidal endothelial cell C-type lectin (LSECtin) (7), galectin-3 (8), and fibrinogen-like protein 1 (FGL1) (9). LAG-3 negatively regulates T cell activation (10), which results in reduced proliferation, decreased cytokine production, diminished cytolytic activity, and impaired memory formation (5, 6, 11). Although a full understanding of the downstream molecular mechanism has yet to be obtained, recent advances have determined that LAG-3 associates with the TCR-CD3 complexes at immunological synapses and induces dissociation of the tyrosine kinase Lck from CD4 or CD8 coreceptors, thereby dampening the magnitude of TCR-induced signaling (12).

Elevated LAG-3 expression on tumor-infiltrating lymphocytes (TILs) has been found broadly in various solid tumor types, and has been significantly associated with unfavorable clinicopathological characteristics (5). Targeting LAG-3 in combination with other checkpoints to enhance antitumor immunity is a rational approach as LAG-3 has often been found co-expressed with PD-1 on tumor-infiltrating T cells in murine models (13–15) and human disease (16–18), and persistent upregulation of both is connected to a state of T cell dysfunction termed exhaustion in models of chronic viral infection (19). Moreover, co-expression of LAG-3 and PD-1 in TIL CD8 T cells has been associated with a greater level of dysfunction than that of cells that express only PD-1 (20, 21). Although the antitumor activity of LAG-3 inhibition alone is modest relative to PD-1 inhibition, combination blockade demonstrates strong synergy in preclinical models (13, 14, 22). Dual PD-1 and LAG-3 blockade was recently approved in advanced or unresectable melanoma in 2022, and there are currently numerous clinical trials evaluating the combination across a diversity of solid tumor types, including non-small cell lung, colorectal, hepatocellular carcinoma, and resected melanoma.

Extensive cellular heterogeneity necessitates the use of comprehensive high parameter immunophenotyping to deeply characterize human immune cells, which may yield actionable translational insights to inform upon target mechanism-of-action, tumor type prioritization, and potential combination strategies. In this study, we utilized mass and flow cytometry to perform high parameter immunophenotyping of immune cells from two independent sample sets representing a diversity of solid tumor types to identify and characterize cell populations that highly express LAG-3 and its ligands. We then used single cell RNA-sequencing (scRNAseq) of circulating immune cells from a clinical trial cohort of melanoma patients treated with immune checkpoint inhibitors (ICI) to explore the potential relationship between LAG-3-expressing cells and clinical outcomes. Our results provide insights into the biology of LAG-3 and its ligands in the TME and have implications for the continued development of dual PD-1- and LAG-3-targeting agents in solid tumors.

## 2 Materials and methods

### 2.1 Sample acquisition

Human tumor and whole blood samples were commercially procured from Avaden BioSciences (WA, USA), BioOptions (CA, USA), Discovery Life Sciences (CA, USA), or MT Group (CA, USA). For the CyTOF cohort, a total of 28 tumor samples from 7 tumors types and 7 matched PBMC samples were profiled. For comparison, PBMCs from 10 healthy donors, isolated from whole blood obtained from the BMS internal blood donor program (n=8) or leukopaks commercially procured from Biospecimen Solutions, Inc. (CA, USA) (n=2) were also included (**Table 1**). For the flow cytometry cohort, a total of 18 tumor samples from 5 tumor types were included (**Table 2**). One sample (TRD-012812) was utilized in both cohorts. All patients gave written informed consent at time of sample collection according to IRB protocols of each provider (23).

**Table 1:** CyTOF cohort specimen metadata and patient demographics.

Patient Identification	Healthy PBMC	Patient PBMC	Patient TIL	Tumor Type	Histology	Stage	T	N	M	Grade	Age	Sex
TRD-001817	N/A	X	X	Kidney	Oncocytoma (benign)	N/A	N/A	N/A	N/A	N/A	63	M
TRD-011400	N/A	X	X	Melanoma	Metastasis (bile duct)	IV	N/A	N/A	N/A	N/A	78	F
TRD-011409	N/A	N/A	X	Gastrointestinal	Gastrointestinal stromal tumor (GIST)	IIB	N/A	N/A	N/A	2	68	M
TRD-011425	N/A	N/A	X	Kidney	Collecting duct carcinoma	N/A	pT3	pNx	N/A	3	81	M
TRD-012134	N/A	N/A	X	Endometrial	Endometrioid carcinoma	IIIC2	pT2	pN2a	N/A	3	67	F
TRD-012194	N/A	X	X	Neuroendocrine carcinoma	Carcinoid	IV	N/A	N/A	N/A	1	45	M
TRD-012236	N/A	N/A	X	Kidney	Clear cell renal cell carcinoma	I	pT1b	pNx	pM0	2	46	M
TRD-012764	N/A	N/A	X	Colon	Mucinous adenocarcinoma	IIC	pT4b	pN0	pM0	2	70	F
TRD-012812	N/A	N/A	X	Endometrial	Adenocarcinoma	IA	pT1a	pNx	pMx	N/A	70	F
TRD-012814	N/A	N/A	X	Kidney	Clear cell renal cell carcinoma	I	pT1b	pNx	pMx	N/A	61	M
TRD-012824	N/A	N/A	X	Colon	N/A	N/A	N/A	N/A	N/A	N/A	N/A	N/A
TRD-012827	N/A	N/A	X	Colon	Adenocarcinoma	pT3	pN0	N/A	3	61	F	N/A
TRD-012832	N/A	N/A	X	Colon	Adenocarcinoma	IV	pT4b	pN2a	pMx	2	64	M
TRD-012868	N/A	N/A	X	Colon	Adenocarcinoma	I	pT2	pN0	pMx	2	63	F
TRD-012880	N/A	N/A	X	Endometrial	Carcinosarcoma	IB	pT1b	pN0	N/A	High	72	F
TRD-012887	N/A	N/A	X	Colon	Mucinous adenocarcinoma	II	pT4b	pN0	pM0	2	44	F
TRD-012895	N/A	N/A	X	Endometrial	Endometrioid carcinoma	I	pT1a	pN0	pM0	N/A	N/A	F
TRD-012897	N/A	X	X	Melanoma	Metastasis (left groin)	IV	pT4b	pN3	pM1b	N/A	87	F
TRD-012924	N/A	N/A	X	Endometrial	Endometrioid carcinoma	IB	pT1b	pN0	N/A	2	57	F
TRD-012955	N/A	X	X	Endometrial	Adenocarcinoma	IIIC1	pT1a	pN1	N/A	1	69	F
TRD-012970	N/A	N/A	X	Colon	N/A	N/A	N/A	N/A	N/A	N/A	52	M
TRD-012975	N/A	X	X	Endometrial	Carcinosarcoma	IA	pT1a	pN0	N/A	1	68	F
TRD-012988	N/A	N/A	X	Colon	N/A	N/A	N/A	N/A	N/A	N/A	45	M
TRD-013012	N/A	N/A	X	Endometrial	Squamous cell carcinoma	I	pT1b	pNx	pMx	2	72	F
TRD-013013	N/A	N/A	X	Colon	Adenocarcinoma	IIB	pT4a	pN0	pM0	2	33	M
TRD-013023	N/A	X	X	Endometrial	Dedifferentiated carcinoma	IB	pT1b	pN0	N/A	N/A	68	F
TRD-013064	N/A	N/A	X	Colon	Adenocarcinoma	I	pT1	pN0	N/A	1	76	F
TRD-013073	N/A	N/A	X	Lung	N/A	N/A	pT2a	pN0	N/A	3	70	F
NHV-000036	X	N/A	N/A	N/A	N/A	N/A	N/A	N/A	N/A	N/A	N/A	M
NHV-000060	X	N/A	N/A	N/A	N/A	N/A	N/A	N/A	N/A	N/A	39	F
NHV-000094	X	N/A	N/A	N/A	N/A	N/A	N/A	N/A	N/A	N/A	N/A	F
NHV-000143	X	N/A	N/A	N/A	N/A	N/A	N/A	N/A	N/A	N/A	N/A	M
NHV-000173	X	N/A	N/A	N/A	N/A	N/A	N/A	N/A	N/A	N/A	N/A	F
NHV-000177	X	N/A	N/A	N/A	N/A	N/A	N/A	N/A	N/A	N/A	N/A	M
NHV-000604	X	N/A	N/A	N/A	N/A	N/A	N/A	N/A	N/A	N/A	N/A	F
NHV-000850	X	N/A	N/A	N/A	N/A	N/A	N/A	N/A	N/A	N/A	N/A	N/A
NHV-001511	X	N/A	N/A	N/A	N/A	N/A	N/A	N/A	N/A	N/A	N/A	N/A
NHV-000851	X	N/A	N/A	N/A	N/A	N/A	N/A	N/A	N/A	N/A	N/A	N/A

**Table 2:** Flow cytometry cohort specimen metadata and patient demographics.

Patient Identification	Patient TIL	Tumor Type	Histology	Stage	T	N	M	Grade	Age	Sex
TRD-012125	X	Colon	Adenocarcinoma	I	T2	N0	Mx	2	89	M
TRD-012730	X	Colon	Adenocarcinoma	IIIA	T2	N1	M0	2	63	M
TRD-012812*	X	Endometrial	Adenocarcinoma	IA	pT1a	pNx	pMx	N/A	70	F
TRD-012101	X	Endometrial	Adenocarcinoma	IIIC1	pT1a	N1a	N/A	1	65	F
TRD-013068	X	Endometrial	Undifferentiated carcinoma	IIIC2	pT1a	N2a	N/A	high	62	F
TRD-013117	X	Endometrial	Adenocarcinoma	II	pT1b	N0	N/A	2	60	F
TRD-012809	X	Liver		N/A	N/A	N/A	N/A	N/A	76	M
TRD-012914	X	Lung	Adenocarcinoma	N/A	pT2b	N0	N/A	2	72	F
204-Y001	X	Lung	Invasive Adenocarcinoma	IA	T1c	N2	M0	3	58	F
TRD-011622	X	Kidney		N/A	N/A	N/A	N/A	N/A	64	M
TRD-011045	X	Kidney	Clear cell renal cell carcinoma	II	pT2a	N/A	N/A	2	73	M
TRD-012144	X	Kidney		N/A	N/A	N/A	N/A	N/A	66	M
TRD-013010	X	Kidney	Papillary Renal Cell Carcinoma	I	T1a	Nx	Mx	2	53	M
TRD-013070	X	Kidney		N/A	N/A	N/A	N/A	N/A	53	M
TRD-013072	X	Kidney		N/A	N/A	N/A	N/A	N/A	70	M
TRD-013134	X	Kidney	Papillary Renal Cancer	N/A	N/A	N/A	mT1a	2	57	M
TRD-013014	X	Lung	Invasive Squamous Cell	IIIA	T4	N0	Mx	N/A	62	M
TRD-013076	X	Colon	Adenocarcinoma	II	T3	N0	M0	2	77	F

### 2.2 Sample processing

Tumor samples and matching blood were received and processed within 24hr of collection, as detailed previously (24). To isolate tumor-infiltrating lymphocytes, tumors were enzymatically digested to generate single-cell suspensions. Briefly, tumors were minced with scalpels in an enzyme cocktail, consisting of 50 U/mL Collagenase I, 17.7 U/mL Collagenase II, 52 U/mL Collagenase IV, 0.1 U/mL Elastase (Worthington Biochemical, Lakewood, NJ), and 0.5 mg/mL DNase I (Sigma-Aldrich, St. Louis, MO). Isolated cells were then cryopreserved in FBS + 10% DMSO until use. On the day of use, TIL samples were thawed in a 37°C water bath for 30 sec, resuspended in 1X CTL Anti-Aggregate Wash Supplement (ImmunoSpot, Cleveland, OH, USA) in serum-free media, incubated for 15min at 37°C, and resuspended in complete medium. Cell viability post-thaw ranged from 29% - 75% as assessed by acridine orange and propidium iodide (AOPI) staining using a Cellaca MX cell counter (Nexcelom Bioscience, Lawrence, MA, USA).

PBMCs were isolated from peripheral blood and leukopaks by density gradient centrifugation and resuspended in FBS + 10% DMSO for cryopreservation until use. For flow cytometry profiling, PBMCs were thawed and stimulated for 3 days with 10µL/mL ImmunoCult™ Human CD3/CD28/CD2 T Cell Activator (StemCell Technologies, Vancouver, Canada) and 50U/mL IL-2 (R&D Systems, Minneapolis, MN, USA) cultured in ImmunoCult™ XF T Cell Expansion Medium (StemCell Technologies) for use as a positive control for marker expression.

### 2.3 Mass cytometry data acquisition

An antibody panel recognizing a total of 37 proteins (18 lineage and 19 target proteins), was used to profile marker expression among the major lymphocyte and myeloid lineages (**Supplementary Table 1**). Additionally, each sample was also stained with a mass-minus-many (MMM) panel consisting of only the 18 lineage markers for background correction. Sample staining and acquisition was performed in a total of 9 batches, with samples from 3 – 4 individuals being included in each batch. To reduce batch effects, subject-matched PBMC and TIL were included in the same batch, and all samples in a batch were uniquely barcoded and pooled prior to acquisition. Each batch included an aliquot of normal healthy PBMC (NHV-000851) to monitor reproducibility among batch runs (**Supplementary Figure 1**).

The staining methods for each batch were as follows: after gentle thawing, samples were first stained with a pre-fix antibody mix containing anti-CD16-209Bi (clone 3G8) and anti-CD112-APC (clone TX31), and then stained for viability with Cell-ID Cisplatin (Fluidigm). Cells were then fixed in 1.5% formaldehyde and stored at 4°C overnight. The next day, samples were aliquoted and washed with ice-cold 0.04% Saponin/PBS and incubated with their unique barcode from the Cell-ID^™^ 20-Plex Pd Barcoding Kit (Fluidigm) in 0.5 mL ice-cold barcoding solution containing 0.04% Saponin/PBS for 15 minutes. Following barcoding, samples were pooled together based on staining panel, and the resulting 2 pools of samples (1 pool for each panel) were blocked with human IgG, stained with a surface antibody cocktail in Maxpar^®^ Cell Staining Buffer (Fluidigm), washed, and then blocked with mouse serum in Perm Buffer (Invitrogen eBioscience). Samples were then stained with an intracellular antibody cocktail in Perm Buffer (Invitrogen eBioscience). The antibody composition of surface and intracellular cocktails is found in **Supplementary Table 1**. Next, the 2 pools were washed in Perm Buffer (Invitrogen eBioscience), combined into 1 pool, and the resulting pool was incubated with 1X Cell-ID™ Intercalator-Ir (Fluidigm) in 2% formaldehyde/PBS overnight at 4°C. The following day, the pool of cells was washed and split into multiple wells of a 96 deep well plate (2 million cells per well). Immediately prior to data acquisition, the cells in each well were adjusted to a cell concentration of 1.0 x 10^6^ cells/mL in 0.1X EQ beads (1 part EQ Four Element Calibration Beads (Fluidigm), 9 part Maxpar^®^ Cell Acquisition Solution (Fluidigm), and were read on a Helios™ CyTOF^®^ mass cytometer (Fluidigm^®^) with WB injector.

### 2.4 Mass cytometry data analysis

Following data acquisition, FCS files generated in the same batch were concatenated, bead-normalization was performed on the concatenated FCS file using Fluidigm CyTOF software, debris was removed by manual gating in FlowJo version 10.6.2, and pooled data was de-barcoded using Fluidigm CyTOF software. De-barcoded FCS files for each sample were then manually gated to remove EQ beads, CD45^-^ cells, doublets, and dead cells (**Supplementary Figure 2A**). New FCS files containing single, viable CD45+ cells were generated for each sample and used for subsequent analysis.

Computational analysis using a combination of nonlinear dimensionality reduction and clustering analysis was performed. First, live, CD45^+^ cells from each sample were down-sampled such that individual samples were equally represented within sample types, and each sample type contained a total of 49,560 events. Next, the down-sampled events were concatenated into a single FCS file, and the opt-SNE implementation of the tSNE algorithm (25) was performed in FlowJo using the default parameters (learning configuration: auto, learning rate: 7% of analyzed events, perplexity: 30, K-nearest neighbors algorithm: Exact (vantage point tree), and gradient algorithm: Barnes-Hut). Cells were then clustered by implementing the FlowSOM v. 2.9 (26) algorithm using the following parameters: 11 metaclusters and a self-organizing map grid size of 10x10. All lineage markers on the CyTOF panel (**Supplementary Table 1**) were used for running the opt-SNE and FlowSOM algorithms, except for CD45 which was excluded. The resulting FCS file was manually gated to export 3 separate FCS files (one for each sample type), and these files were imported into OMIQ software from Dotmatics (www.omiq.ai, www.dotmatics.com) for data visualization. FlowSOM metaclusters were overlaid on the t-SNE maps, and the metaclusters were annotated by visual inspection of marker expression within each metacluster on the t-SNE map. Manual merging of highly similar metaclusters was performed following r^2^ correlation analysis and consideration of biological relevance. Findings from this analysis were confirmed by calculating percent positive frequencies of a similar set of 10 broad lymphocyte and myeloid populations identified by manual gating in FlowJo. Manual gating was also used to determine frequencies of T cell subsets and the percent positive frequency of LAG-3 within each subset (**Supplementary Figure 2A, B**).

A similar machine learning approach was employed to visualize co-expression of various markers among T cell metaclusters and to identify T cell metaclusters with the highest expression intensity of LAG-3. Live CD45^+^/CD3^+^/CD14^-^/CD15^-^ T cells from each sample were manually gated (**Supplementary Figure 2A, B**), and equal down-sampling of the T cell population was performed, resulting in each sample type containing a total of 14,308 events. The opt-SNE algorithm (25) was performed using default parameters, and FlowSOM (26) was run using 10 metaclusters and a self-organizing map grid size of 10X10. Additionally, for running both the opt-SNE (25) and FlowSOM (26) algorithms, twenty two markers known to be expressed on T cells (4-1BB, CCR7, CD112R, CD226, CD25, CD27, CD28, CD4, CD45RO, CD73, CD8, CD96, Eomes, FOXP3, HLA-DR, ICOS, Ki67, LAG-3, PD-1, Tbet, TIGIT, and TIM-3) were used.

To quantify the co-expression of LAG-3 and PD-1 among CD8 TCM and TEM cell subsets in TIL samples, percent positive frequencies of PD1^-^/LAG3^-^, PD1^-^/LAG3^+^, PD1^+^/LAG3^-^, and PD1^+^/LAG3^+^ subsets of CD8 TCM and TEM T cells were calculated in FlowJo using Boolean gating after performing the manual gating described in **Supplementary Figures 2A, B**. Expression/co-expression of target markers in these TIL CD8 TCM and TEM PD1/LAG3 subsets was quantified by both arcsinh-transformed (co-factor of 5) median ion count signal intensity and percent positive frequency. Percent positive frequencies were derived from Boolean gating after performing the manual gating described in **Supplementary Figures 2A, B**.

### 2.5 Flow cytometry data acquisition

Two high-parameter flow cytometry panels (21 fluorophores each) were developed to characterize immune cells expressing LAG-3 and its ligands, focusing on T cells (T cell-focused panel; **Supplementary Table 2**) and broad immune lineages (T/B/NK/Myeloid panel; **Supplementary Table 3**), respectively. In order to define positive and negative gates, fluorescence minus one (FMO) samples were included for the respective T cell checkpoint markers and LAG-3 ligands. All samples were stained and acquired in a single experiment to avoid batch effects.

The staining methods were as follows: The T cell-focused panel included staining with a pre-fix antibody CCR7 (BV605), while the T/B/NK/Myeloid panel was first stained with 5µL of BD Human Fc IgG block to prevent nonspecific staining. Cells were sequentially stained for viability and then surface markers, fixed and permeabilized using the Foxp3 Transcription Factor Staining Buffer Set (ThermoFisher Scientific), and stained for intracellular markers. All samples were then fixed with 2% PFA and stored at 4°C until acquisition on the cytometer. Sample data were acquired using a BD FACSymphony A5 flow cytometer (BD Biosciences, San Jose, CA) and data analysis was performed in FlowJo (Version 10.6.2) using the manual gating strategy depicted in **Supplementary Figures 3 and 4**.

### 2.6 Unsupervised analysis of T cell flow cytometry

FlowAI (27) was used to clean flow cytometry data for unsupervised analysis, followed by manual gating to exclude duplets, a time gate was used to further clean data, and manually gating was performed to select for live CD45^+^CD3^+^CD19^-^ T cells. T cells were equally down-sampled to 1,453 T cells per sample. Fluorescence intensity values were transformed using arcsinh transformation with co-factors manually set for each channel as described by Melson et al. (28). The opt-SNE algorithm (25) was performed using default parameters, and FlowSOM (26) was run using 14 metaclusters and a self-organizing map grid size of 10X10. One metacluster with a total of 47 events (most of which were from a single sample), was deemed an artifact and excluded. All markers on the T cell flow cytometry panel (**Supplementary Table 2**), except for CD45, CD3, and CD19, were used to run both the opt-SNE (25) and FlowSOM (26) algorithms.

### 2.7 Clinical cohort

Peripheral blood samples for scRNAseq were obtained from patients enrolled in the phase 2 Checkmate 069 trial (NCT01927419) for patients with previously untreated, unresectable stage III or IV melanoma. Details of the study design and trial results have been published previously (29, 30). Briefly, patients were randomized 2:1 to receive nivolumab 1 mg/kg plus ipilimumab 3 mg/kg or ipilimumab 3 mg/kg plus placebo, every 3 weeks for four doses. Subsequently, patients assigned to nivolumab plus ipilimumab received nivolumab 3 mg/kg every 2 weeks until disease progression or unacceptable toxicity, whereas patients allocated to ipilimumab alone received placebo every 2 weeks during this phase. Response was determined by Response Evaluation Criteria in Solid Tumors (RECIST), version 1.1. Patients from the nivolumab plus ipilimumab arm were profiled for best overall response, with 15 patients in the complete responder (CR), 8 patients in the progressive disease (PD) and 3 patients in the stable disease (SD) subgroups.

### 2.8 Single cell RNA-sequencing

Banked patient PBMCs from either Baseline (cycle 1, day 1; C1D1) or on treatment (cycle 3, day 1; C3D1) were thawed, washed to remove serum and dead cells, and resuspended in PBS with 0.04% BSA for emulsion. Single cell suspensions targeting 10^4^ cells/sample were generated with the Chromium Next Gen 5’ V2 kit from 10x Genomics (Pleasanton, CA, USA). cDNA and libraries were generated according to the 10x Genomics published user guide protocol (CG000331). Libraries were sequenced to target 100,000 reads/cells. The experiment yielded 816,122 profiled cells across 100 samples with an average of 8,161 cells/sample and median 1,500 genes/cell post filtering.

To analyze the scRNAseq dataset, filtered count matrices were generated using the 10X Genomics cellranger count pipeline and normalization per sample using SCTransform. Cell type allocation was determined using the SingleR Bioconductor package and the Blueprint/ENCODE reference. The sample data was then queried for count and feature distributions and filtered to remove cells that had >25% mitochondrial gene transcripts and <200 or >10,000 genes expressed. The samples were then integrated using the Harmony package and annotated for downstream analysis in R (v. 4.2.1) using the Seurat package (v. 4.1.1) (31–34; 35).

To visualize the multi-dimensional data, the function “RunPCA” computed principal components (PCs) on the integrated assay, the first 30 of which were selected, based on the Seurat elbow plot, and specified as the dims argument to the “FindNeighbors” and “RunUMAP” functions. UMAP plots were then used to visualize cell type distributions and feature distributions. To look at expression distributions for selected genes we used dot plots and violin plots via Seurat. ANOVA p-values were furnished in Violin plots using the ggpubr R package.

### 2.9 Statistical analysis

For mass cytometry and flow cytometry analysis, Mann-Whitney tests were performed in Prism version 8.4.1 (GraphPad) to determine statistically significant differences in arcsinh-transformed median ion count (mass cytometry) or median fluorescence (flow cytometry) intensities and target percent positive frequencies among TIL subsets. For multiple comparisons, Bonferroni correction was applied to adjust raw p values. For all analyses, p <0.05 was considered statistically significant. Sample populations with <150 events were excluded from analysis to reduce potential spurious artifacts.

For scRNAseq analysis, ANOVA p-values were furnished in Violin plots using the ggpubr R package. Differential gene expression was assessed using the Wilcoxon rank sum test (as implemented by FindMarkers in Seurat). Genes with an adjusted p-value (based on Bonferroni correction) < 0.05 were considered significantly differentially expressed.

## 3 Results

### 3.1 Characterization of peripheral and tumor infiltrating immune cell populations using mass cytometry

High parameter immunophenotyping was used to broadly characterize various immune cell types from patients with cancer. We accomplished this using cytometry by time-of-flight (CyTOF) to analyze marker expression from a commercial cohort comprised of seven different solid tumor types. These commercially obtained samples consisted of 28 TILs isolated from fresh tumor tissue, with matched PBMCs from 7 of the patients. PBMCs from 10 healthy donors were also included for comparison. These samples were derived from 7 different solid tumor types that included a range of stages and grades to ensure broader applicability of profiling results. (**Table 1**).

**Figure 1:**
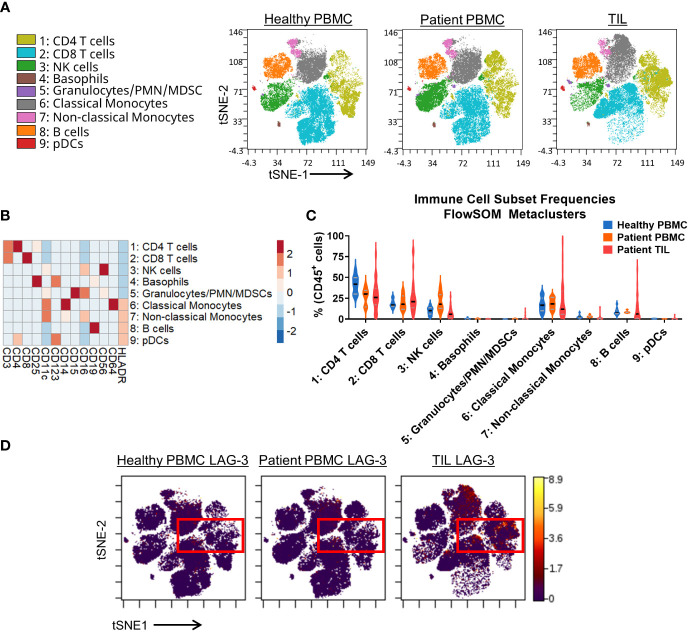
Broad immunophenotyping and LAG-3 expression profiling in the CyTOF cohort. **(A)** FlowSOM-overlaid tSNE maps for healthy PBMC (n=10), patient PBMC (n=7), and patient TIL (n=28) samples that were generated using the opt-SNE dimensionality reduction and FlowSOM v2.9 clustering algorithms. NK, natural killer; PMN MDSC, polymorphonuclear myeloid-derived suppressor cell; pDC, plasmacytoid dendritic cell. **(B)** Heatmap depiction of z-score normalized, arcsinh-transformed (co-factor of 5) median ion count signal intensity values for the indicated lineage markers among metaclusters identified in **(A)** from all samples. **(C)** Frequencies of the FlowSOM-derived metaclusters identified in **(A)** from all samples. Black and gray lines denote median and quartile values, respectively. **(D)** Localization of LAG-3 signal intensity on the t-SNE maps generated in **(A)**. Red box denotes the regions with the highest expression of LAG-3.

To generate a broad and comprehensive view of the immune compartment, we utilized the opt-SNE (25) dimensionality reduction algorithm, which is based on t-distributed stochastic neighbor embedding (t-SNE), to generate two-dimensional maps of CD45+ cells from all sample types. The data were then clustered into distinct immune cell types using the FlowSOM algorithm (26) and overlaid onto the t-SNE maps. For both algorithms, only the lineage markers listed in **Supplementary Table 1** were considered to identify 9 major immune cell lineages (**Figures 1A-C**). In parallel, we also used manual gating based on immune lineage marker expression to identify a similar set of 10 broad lymphocyte and myeloid lineages and 11 distinct subsets of T cells (**Supplementary Figures 5A, B**). The composition of broad immune cell lineages was similar between the healthy donor PBMCs, cancer patient PBMCs, and TIL samples (**Figure 1C**; **Supplementary Figure 5A**). However, T cell subsets among TIL samples showed an expected enrichment of the median frequency of CD4 TEM cells (19.7% vs. 10.6 - 10.8%), CD8 TEM cells (24.0% vs. 5.4 - 7.4%), and Tregs (8.9% vs. 1.7 - 2.5%), and reduction of naïve T cells (0.9% vs. 18.3 - 38.4%; **Supplementary Figure 5B**) compared to peripheral T cells from heathy donors and cancer patients.

### 3.2 CD8 memory T cells were the predominant LAG-3^+^ immune cell type in human solid tumors

Our CyTOF investigation utilized a single LAG-3 antibody for total staining (surface and intracellular). The tSNE analysis showed that the LAG-3 expression was enriched in TILs compared to PBMCs from either healthy donors or cancer patients (**Figure 1D**). Among the TILs, the greatest levels of LAG-3 expression were observed within the CD4 and CD8 T cells, with some expression noted in classical monocytes (**Figures 1A, D**). Similarly, manual gating showed the highest median frequency of LAG-3 expression on TIL CD8 TEM (18.2%) and TCM (13.5%) T cells (**Supplementary Figure 5C**). In contrast, among the CD4 T cell subsets, the highest median frequency of LAG-3 expression was observed on the CD4 Th EM cells (6.8%) and Tregs (5.7%; **Supplementary Figure 5C**). When we assessed LAG-3 expression in each subset as a proportion of the total tumor infiltrating T cells, the CD8 TEM cells were found to constitute the bulk of LAG-3^+^ T cells within the tumor microenvironment, with a median frequency of 3.6% (range = 0.2% – 32.9%; **Supplementary Figure 5D**). Notably, these LAG-3^+^ CD8 TEM cells accounted for ≥10% of the total tumor-infiltrating T cells in one quarter (7/28) of the samples. CD4 TEM cells were the next highest frequency LAG-3^+^ T cell subset, with a median frequency of 1.5% (range = 0.1% - 6.1%). All other subsets exhibited a lower median frequency (≤ 0.6%) and a lower upper limit to their range (≤ 8.4%; **Supplementary Figure 5D**). Taken together, these results confirm that most of the LAG-3 expression in patients with solid tumors is found on tumor infiltrating, but not circulating, CD8 and CD4 memory T cells.

### 3.3 LAG-3 expression was associated with a unique marker profile among solid tumor-infiltrating T cells

**Figure 2:**
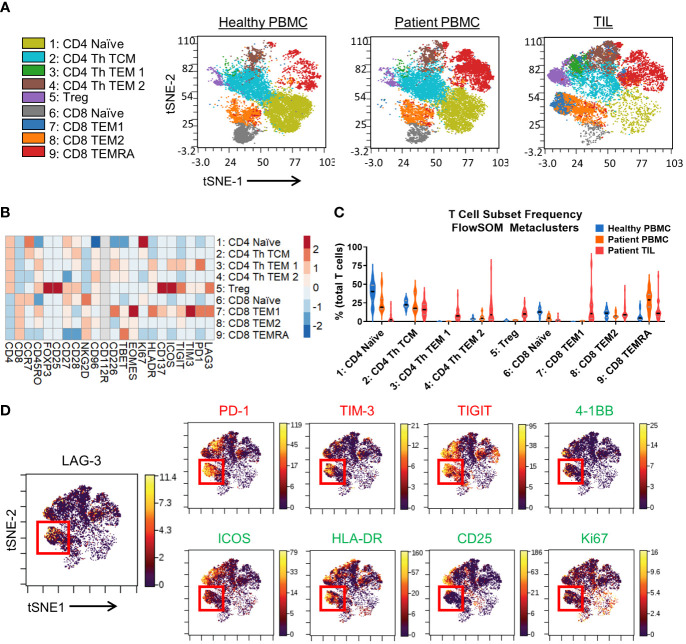
T cell immunophenotyping and LAG-3 expression profiling in the CyTOF cohort. **(A)** FlowSOM-overlaid t-SNE maps for healthy PBMCs (n=10), patient PBMCs (n=7), and patient TIL (n=28) samples generated using the opt-SNE dimensionality reduction and FlowSOM clustering algorithms. **(B)** Heatmap depiction of z-score normalized, arcsinh-transformed (co-factor of 5) median ion count signal intensity values for the indicated T cell markers among metaclusters identified in **(A)** from patient TIL samples. **(C)** Frequencies of the FlowSOM-derived T cell metaclusters as a percentage of total T cells from all samples. Black and gray lines denote median and quartile values, respectively. **(D)** Left, localization of LAG-3 signal intensity on the patient TIL t-SNE map generated in **(A)**. Red box denotes the region with the highest expression of LAG-3. Right, colocalization of the indicated inhibitory, stimulatory, and activation markers with LAG-3 signal intensity on the patient TIL t-SNE map generated in **(A)**. Red box denotes the overlapping region of highest LAG-3 expression. .

Since we observed enrichment of LAG-3 expression primarily among T cells, we sought to employ further nonlinear dimensionality reduction and clustering analysis of only the T cell compartment to gain a global view of the marker profile of LAG-3-expressing cells. This analysis resulted in 9 distinct metaclusters of T cell subsets based on the expression of 22 markers (**Figures 2A , B**). Like manual gating analysis, the frequencies of specific metaclusters differed among TILs vs. peripheral samples (**Figure 2C**). In particular, the metaclusters 7 (CD8 TEM 1) and 3 (CD4 Th TEM) were only found in the TILs (**Figure 2A, C**).

Analysis of LAG-3 signal intensity revealed substantial expression in metaclusters 7 (CD8 TEM 1) and 5 (Treg), and limited expression in the other metaclusters (**Figure 2B**). Indeed, visualization of LAG-3 in the TIL T cell tSNE map (**Figure 2D**) confirmed the most enrichment of LAG-3 within a focal region of metacluster 7. Other markers expressed at a relatively high intensity by metacluster 7 included the checkpoints PD-1, TIM-3, and TIGIT, the activation marker HLA-DR, as well as the costimulatory receptors 4-1BB and ICOS (**Figures 2B, D**). Notably, compared to the more tightly regionally localized LAG-3 signal, these markers exhibited more broadly distributed expression profiles across other metaclusters (**Figure 2D**). Metacluster 7 also displayed a higher median signal intensity for the Eomes transcription factor versus the Tbet transcription factor (**Figure 2B**), which has been associated with T cell exhaustion and dysfunction in chronic infection (36, 37) and cancer (38, 39), respectively. We also evaluated the activation marker CD25 and the proliferation marker Ki67 and observed only a partial overlap with the LAG-3 signal in metacluster 7 (**Figure 2B, D**). Taken together, these results indicate that LAG-3 expression may discriminate a subset of TIL CD8 memory T cells with a unique marker expression profile characterized by simultaneous expression of multiple inhibitory and stimulatory receptors.

### 3.4 PD-1 was highly co-expressed by TIL LAG-3^+^ CD8 memory T cells

**Figure 3:**
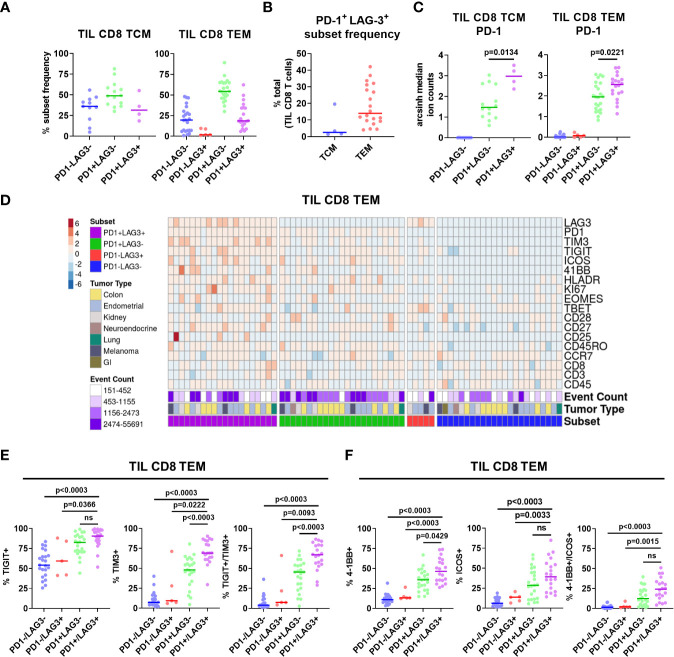
Characterization of PD-1/LAG-3 subsets of TIL CD8 memory cells in the CyTOF cohort. **(A)** Frequencies of PD-1/LAG-3 subsets in TIL CD8 TCM (left) and TEM (right) cells. CD8 TCM and TEM populations were manually gated, and Boolean gating was performed from those populations to derive PD-1/LAG-3 subsets of each. Sample populations with <150 events were excluded from the analysis. Lines indicate median values. **(B)** Frequencies of PD-1^+^ LAG-3^+^ TCM and TEM subsets as a percentage of total TIL CD8 T cells. Sample populations with <150 events were excluded from the analysis. Lines indicate median values. **(C)** Arcsinh (co-factor of 5)-transformed median ion count signal intensities of PD-1 among TIL CD8 TCM (left) and TEM (right) cells. Mann-Whitney tests were performed to determine statistically significant differences in PD-1 signal intensity. Bonferroni adjusted p values < 0.05 were considered statistically significant. Sample populations with <150 events were excluded from the analysis. Lines indicate median values. **(D)** Heatmap depictions of z-score normalized, arcsinh-transformed (co-factor of 5) median ion count signal intensity values for the indicated markers among PD1/LAG3 subsets of TIL CD8 TEM cells. Each column represents a different sample and is annotated with PD-1/LAG-3 subset, tumor type, and event count. Colors are as indicated. Sample populations with <150 events were excluded from the analysis. **(E)** Frequencies of TIGIT, TIM-3, and TIGIT + TIM-3 co-expression among the TIL CD8 TEM PD-1/LAG-3 subsets. **(F)** Frequencies of 4-1BB, ICOS, and 4-1BB + ICOS co-expression among PD-1/LAG-3 subsets of TIL CD8 TEM cells. Boolean gating was performed to determine TIGIT, TIM-3, 4-1BB, and ICOS expression/co-expression in each subset. To determine statistically significant differences among subsets, Mann-Whitney tests were performed, and p values were adjusted using Bonferroni correction. Adjusted p values <0.05 were considered statistically significant. Sample populations with <150 events were excluded from the analysis. Lines indicate median values.

Given the apparent regional co-localization of LAG-3 with other inhibitory receptors, we utilized additional manual gating to confirm these findings by first profiling the frequency of PD-1 and LAG-3 co-expression in CD8 memory T cells. Representative flow plots are shown in **Supplementary Figure 6A**, in which four different PD-1/LAG-3 subsets could be identified in TIL samples from 6 of the 7 tumor types included in this study. By using Boolean gating, we found that the majority of both CD8 TCM and TEM cells were positive for only PD-1 (median frequencies of 49.0% and 54.4%, respectively), but a sizeable proportion were found to be PD-1^+^ and LAG-3^+^ (median frequencies of 31.5% and 18.65%, respectively; **Figure 3A**). This double positive subset was found in 4/28 TIL samples from CD8 CM cells (2/9 endometrial and 2/2 melanoma), and 20/28 TIL samples from the CD8 EM cells (7/10 colon, 8/9 endometrial, 2/4 kidney, 1/1 lung, and 2/2 melanoma). Interestingly, there was a near total lack of cells that only expressed LAG-3 but not PD-1 (**Figure 3A**). When we analyzed the PD-1^+^ LAG-3^+^ CD8 TCM and TEM subsets as a proportion of the total tumor infiltrating CD8 T cells, we found that the double positive TEM cells constituted a sizeable minority of the population, with a median frequency of 14.0% (range = 4.1% – 42.1%; **Figure 3B**). Furthermore, median PD-1 expression intensity was significantly greater in the PD-1^+^ LAG-3^+^ subset vs. the PD-1^+^ LAG-3^-^ subset from both CD8 TCM and TEM cells (**Figure 3C**). Altogether, these data indicate that the majority of LAG-3^+^ CD8 memory T cells highly co-express PD-1.

### 3.5 Tumor infiltrating PD-1^+^ LAG-3^+^ CD8 memory T cells highly co-expressed multiple inhibitory and stimulatory checkpoints

After separating the CD8 TCM and TEM cells into subsets based on PD-1 and LAG-3 presence, we then profiled these subsets for the co-expression of additional markers. Marker signal intensity profiles of the PD-1^+^ LAG-3^+^ subset from both CD8 TCM and TEM were distinct from that of the profiles of cells that expressed either PD-1 or LAG-3, or were negative for both, which was observed across tumor types (**Supplementary Figure 7A**; **Figure 3D**). Manual gating was used to confirm marker co-expression (**Supplementary Figures 6B–E**). Analysis of the expression of the inhibitory receptors TIGIT and TIM-3 revealed that the PD-1^+^ LAG-3^+^ subsets from both CD8 TCM and TEM cells also exhibited the greatest expression of each marker, alone and in combination, compared to the subsets that possessed either PD-1 or LAG-3, or were negative for both (**Supplementary Figure 7B**; **Figure 3E**). Similarly, analysis of the expression of the co-stimulatory receptors 4-1BB and ICOS also revealed that the PD-1^+^ LAG-3^+^ CD8 TCM subset expressed more ICOS, and the CD8 TEM subset expressed more 4-1BB (**Supplementary Figure 7C**; **Figure 3F**). When assessed together, we found that 4-1BB and ICOS were co-expressed at a significantly higher frequency in the PD-1^+^ LAG-3^+^ TCM subset (**Supplementary Figure 7C**). We also found significantly higher expression of Ki67 and HLA-DR, but not CD25, in the PD-1^+^ LAG-3^+^ CD8 TCM subset, but not the CD8 TEM subset (**Supplementary Figures 8A, B**). Altogether, these data demonstrate that PD-1^+^ LAG-3^+^ CD8 memory T cells highly co-express additional inhibitory (TIGIT, TIM-3) and stimulatory (4-1BB, ICOS) receptors, and that their marker profile is distinct compared to the cells that express either PD-1 or LAG-3, or are negative for both.

### 3.6 Characterization of an independent cohort of TILs by flow cytometry confirmed the unique marker profile of PD-1^+^ LAG-3^+^ CD8 memory T cells

**Figure 4:**
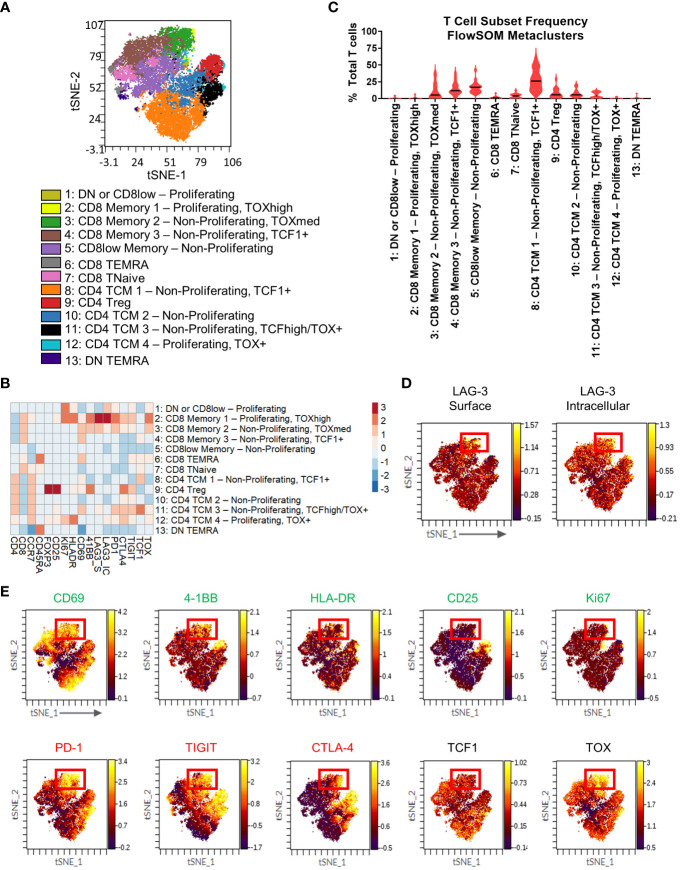
T cell immunophenotyping and LAG-3 expression profiling in the flow cytometry cohort. **(A)** FlowSOM-overlaid tSNE maps for patient TIL (n=18) samples generated using the opt-SNE dimensionality reduction and FlowSOM clustering algorithms. **(B)** Heatmap depiction of z-score normalized, arcsinh-transformed median fluorescence signal intensity values for the indicated T cell markers among metaclusters identified in **(A)** from patient TIL samples. **(C)** Frequencies of the FlowSOM-derived T cell metaclusters as a percentage of total T cells from all samples. Black and gray lines denote median and quartile values, respectively. **(D)** Localization of surface (left) and intracellular (right) LAG-3 signal intensity on the patient TIL tSNE map generated in **(A)**. Red box denotes the region with the highest expression of LAG-3. **(E)** Colocalization of the indicated markers with LAG-3 signal intensity on the patient TIL tSNE map generated in **(A)**. Red box denotes the overlapping region of highest LAG-3 expression. Green text indicates activation-related markers, red text indicates inhibitory markers, and black text indicates transcription factors.

Next, we sought to broaden our investigation by profiling tumor infiltrating T cells from an independent commercially-obtained patient cohort of 18 TIL samples from 5 different tumor types from various stages and grades to ensure broad comparability with the CyTOF cohort. (**Table 2**). Samples were profiled by high parameter flow cytometry using a panel of 21 markers focused on T cells (**Supplementary Table 2**). This panel had some changes from the mass cytometry panel, with the addition of CD69, CTLA-4, TCF1, and TOX, and removal of ICOS, TIM-3, Tbet and Eomes. We employed further nonlinear dimensionality reduction and clustering analysis from only the T cell compartment, which yielded 13 distinct metaclusters of T cell subsets (**Figure 4A, B**). The clustering analysis showed substantial heterogeneity within the CD4 and CD8 T cell subsets, with 4 discrete subsets each of CD4 TCM cells and CD8 memory cells, respectively (**Figure 4A, B**). These CD8 subsets appeared to be a mixture of both TCM and TEM cells (negative for CD45RA expression and variable CCR7 expression) and may reflect a range of exhausted/dysfunctional differentiation states based on their respective variable expression of the transcription factor TOX (40–42). The highest median frequency subsets included metaclusters 8 (CD4 TCM 1 - Non-Proliferating, TCF1^+^; 25.99%), 5 (CD8^low^ Memory - Non-Proliferating; 16.72%), and 4 (CD8 memory 3 - Non-Proliferating, TCF1^+^; 11.77%; **Figure 4C**).

To gain additional insights into LAG-3 expression characteristics, we profiled our flow cytometry cohort samples using antibodies that could discriminate surface LAG-3 and intracellular LAG-3. When we profiled LAG-3 signal intensity in the T cell metaclusters, we observed the highest expression of surface LAG-3 expression in a region of the tSNE map corresponding to two CD8 memory subsets that could be defined by differential expression of Ki67 and TOX (metaclusters 2: CD8 Memory 1 - Proliferating, TOX^high^ and 3: CD8 Memory 2 - Non-Proliferating, TOX^med^; **Figure 4B, D**). These subsets represented a median frequency of 0.10% (range = 0% - 6.47%) and 5.23% (range = 0.21% - 37.85%), respectively, of the total TIL T cell population (**Figure 4C**). Some surface LAG-3 expression was also observed in metacluster 11 (CD4 TCM 3 - Non-Proliferating TCF1^+^ TOX^+^). Intracellular LAG-3 expression was the highest in metaclusters 2 (CD8 Memory 1 - Proliferating, TOX^high^) and 1 (DN or CD8^low^ - Proliferating), with lower expression observed in metaclusters 3 (CD8 Memory 2 - Non-Proliferating, TOX^med^) and 12 (CD4 TCM 4 - Proliferating, TOX^+^ and 1; **Figure 4B**). Interestingly, metacluster 2 expressed the highest intensity of both surface and intracellular LAG-3, but the other subsets showed expression of either surface or intracellular LAG-3, but not both (**Figure 4B, D**). Other markers expressed at a relatively high intensity by the two highest surface LAG-3-expressing CD8 T cell metaclusters included other checkpoints (PD-1, CTLA-4, and TIGIT) and the costimulatory receptor 4-1BB (**Figure 4B**), which agrees with our CyTOF analysis (**Figure 2B**). These metaclusters also displayed the highest intensity expression of TOX, which indicates a dysfunctional differentiation state (43). Assessment of other markers on the tSNE map showed apparent co-localization of this LAG-3^+^ region with several activation, stimulatory, and inhibitory markers (CD69, 4-1BB, HLA-DR, PD-1, TIGIT, CTLA-4, TOX) and a relative dearth of other markers (CD25, TCF1; **Figure 4E**). Notably, the LAG-3 surface signal was more tightly regionally localized compared to the LAG-3 intracellular signal (**Figure 4D**). Most other markers, including all checkpoints assessed here, exhibited more broadly distributed expression profiles across other areas of the tSNE map compared to the LAG-3 surface signal (**Figure 4E**).

**Figure 5:**
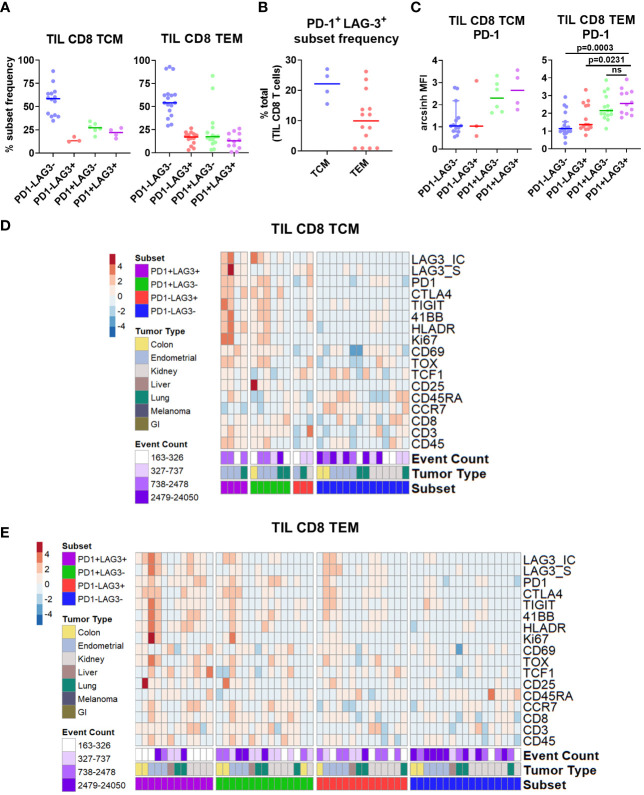
Characterization of PD-1/LAG-3 subsets of TIL CD8 memory cells in the flow cytometry cohort. **(A)** Frequencies of PD-1/LAG-3 subsets in TIL CD8 TCM (left) and TEM (right) cells. CD8 TCM and TEM populations were manually gated, and Boolean gating was performed from those populations to derive PD-1/LAG-3 subsets of each. Sample populations with <150 events were excluded from the analysis. Lines indicate median values. **(B)** Frequencies of PD-1^+^ LAG-3^+^ TCM and TEM subsets as a percentage of total TIL CD8 T cells. Sample populations with <150 events were excluded from the analysis. Lines indicate median values. **(C)** Arcsinh -transformed median fluorescence signal intensity of PD-1 among TIL CD8 TCM (left) and TEM (right) cells. Mann-Whitney tests were performed to determine statistically significant differences in PD-1 signal intensity. Bonferroni adjusted p values < 0.05 were considered statistically significant. Samples population with <150 events were excluded from the analysis. Lines indicate median values. **(D)** Heatmap depictions of z-score normalized, arcsinh-transformed median fluorescence signal intensity values for the indicated markers among PD1/LAG3 subsets of TIL CD8 TCM cells. Each column represents a different sample and is annotated with PD-1/LAG-3 subset, tumor type, and event count. Colors are as indicated. Sample populations with <150 events were excluded from the analysis. **(E)** Heatmap depictions of z-score normalized, arcsinh-transformed median fluorescence signal intensity values for the indicated markers among PD1/LAG3 subsets of TIL CD8 TEM cells. Each column represents a different sample and is annotated with PD-1/LAG-3 subset, tumor type, and event count. Colors are as indicated. Sample populations with <150 events were excluded from the analysis.

Next, we used manual gating in our flow cytometry cohort (**Supplementary Figures 3A, B**) to select the four different PD-1/LAG-3 subsets (**Supplementary Figure 6F**), finding that the majority of both CD8 TCM (CCR7^+^ CD45RA^-^) and TEM (CCR7^-^ CD45RA^-^) cells were positive for only PD-1 (median frequencies of 27.2% and 17.4%, respectively), but a sizeable proportion were found to be both PD-1^+^ and LAG-3^+^ (median frequencies of 22.2% and 12.9%, respectively; **Figure 5A**). This double positive subset was found in 4/18 TIL samples from CD8 TCM cells (3/4 endometrial and 1/3 lung), and 12/18 TIL samples from the CD8 TEM cells (2/3 colon, 3/4 endometrial, 4/7 kidney, 1/1 liver, and 2/3 lung). The double positive subset of CD8 TCM and TEM cells was once again a sizeable minority of the total TIL CD8 population, with a median frequency of 22.2% and 9.9%, respectively (**Figure 5B**). Median PD-1 expression intensity was equivalent between the double positive subsets and the PD-1^+^ LAG-3^-^ subsets from both CD8 TCM and TEM cells (**Figure 5C**). We then profiled these subsets for the co-expression of additional markers. As shown in **Figures 5D, E**, the marker profiles of both CD8 TCM and TEM cells that co-expressed both PD-1 and LAG-3 exhibited generally robust signal intensities for several activation, stimulatory, and inhibitory markers, which was observed across differing tumor types. The median marker intensities were largely equivalent between the PD-1^+^ LAG-3^+^ subsets compared to the PD-1^+^ LAG-3^-^ subsets. Taken together, these results appear to be consistent with the results from our CyTOF cohort and indicate that co-expression of PD-1 and LAG-3 in tumor infiltrating CD8 T cells could be used as a simple way to detect and/or enrich for cells with this unique marker profile suggestive of T cell dysfunction/exhaustion.

### 3.7 Characterization of LAG-3 ligand expression among immune cells from human solid tumor TIL

**Figure 6:**
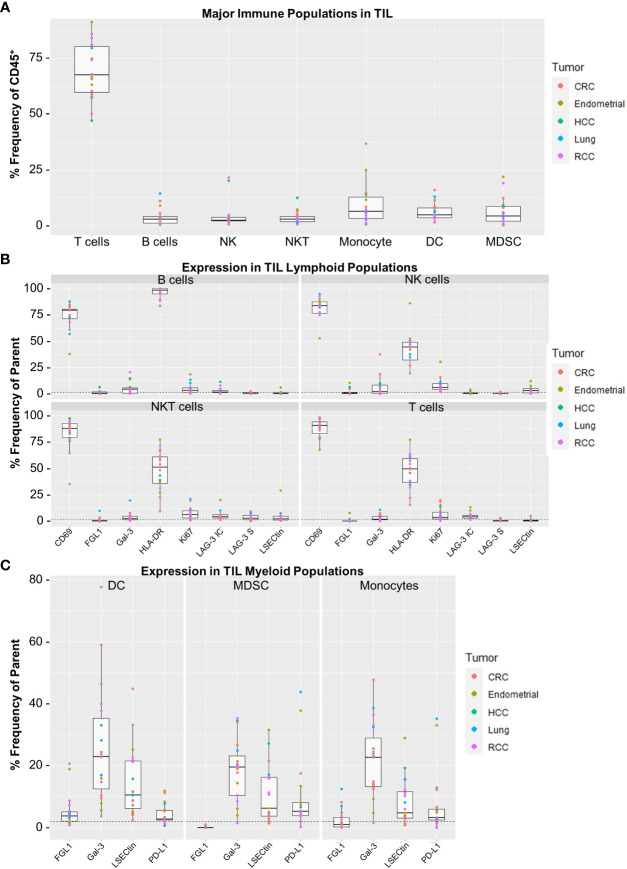
Expression profiling for surface LAG-3, intracellular LAG-3, and LAG-3 ligands in the flow cytometry cohort. **(A)** Frequencies of major TIL immune cell populations. Immune cell populations were defined by manual gating as determined in **Supplemental Figure 4**. Reported values are expressed as Frequency (%) of CD45+ cells for each population. **(B)** Target expression from the TIL lymphocyte populations. Shown are panels for B Cells, NK Cells, NKT Cells and T Cells. **(C)** Target expression from the TIL myeloid populations. Shown are panels for Dendritic Cells, Myeloid Derived Suppressor Cells (MDSC) and Monocytes. Gal-3, galectin-3; LAG3-S, surface LAG-3 expression; LAG3-IC, intracellular LAG-3 expression; NKT, natural killer T cell. Sample populations with <150 events were excluded from the analysis. All boxplot visualizations show the median, first and third quartiles as well as 1.5* IQR (interquartile range). Colored dots indicate individual samples and their corresponding tumor type. Positive gates were determined using control samples stained without the respective target markers and a frequency cut-off of 2% (dashed lines).

Finally, we applied a separate flow cytometry panel of 21 markers for broader immunophenotyping of TILs for the expression of LAG-3 and its ligands (**Supplementary Table 3**). We used manual gating based on immune lineage marker expression to identify a set of 7 broad lymphocyte and myeloid lineages (**Supplementary Figures 4A, B**). As shown in **Figure 6A**, the highest median frequency TIL cell type by far were T cells (67.5%), in agreement with the results observed in the CyTOF cohort (**Supplementary Figure 5A**). Minimal LAG-3 expression was observed in the broad lymphoid populations, with T cells and NKT cells expressing the highest median frequencies of intracellular LAG-3 (both 4.3%) and NKT cells expressing the highest median frequency of surface LAG-3 (2.9%; **Figure 6B**). Neither intracellular nor surface LAG-3 was detected in myeloid cell populations (data not shown). Assessment of LAG-3 ligands included surface detection for HLA-DR and LSECtin, and intracellular detection of galectin-3 and FGL1 because these are secreted proteins. As shown in **Figure 6B**, lymphoid populations widely expressed HLA-DR (median frequency of ≥44.5% for all populations), whereas the other ligands were only minimally expressed (median frequency of <5% in all populations for FGL1, galectin-3, and LSECtin; **Figure 6B**). LAG-3 ligand expression was more abundant in myeloid cells, as all DCs and monocytes were HLA-DR^+^ (gating factor), and all populations expressed galectin-3 at a median frequency of 19.5% - 22.9% (**Figure 6C**). Expression of LSECtin was also observed in a minority of all myeloid populations (median frequency of 4.7% - 10.5%), as was FGL1 in DCs (median frequency 3.8%, range = 0.8 - 20.6%; **Figure 6C**). For comparison, PD-L1 was found at relatively low levels, with a median frequency of ≤ 5.3% for all myeloid populations. As expected, target MFI values for all markers except FGL1 were positively correlated with expression frequency across the various immune cell populations (**Supplementary Figure 9**). This result for FGL1 is likely due to the very low expression observed for this marker.

### 3.8 Elevated *LAG3* transcript expression in peripheral CD8 memory T cells was associated with disease progression in melanoma patients treated with combination immune checkpoint inhibition

**Figure 7:**
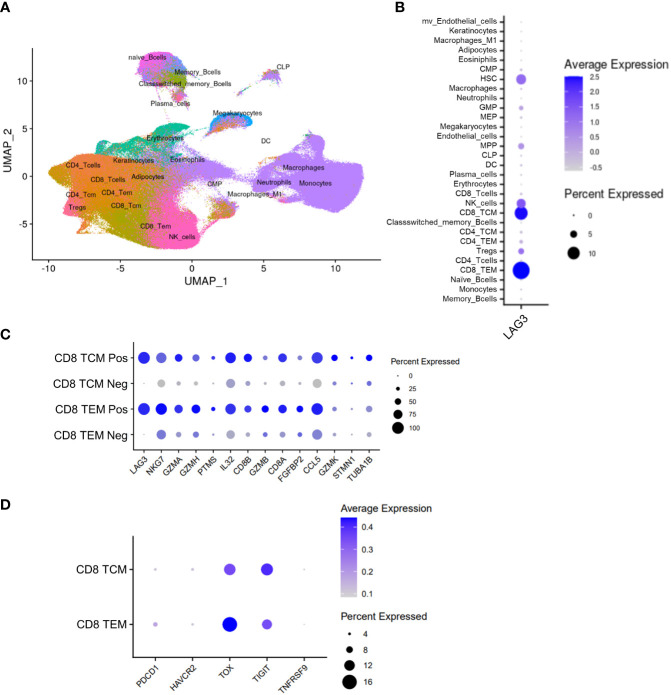
Peripheral scRNAseq and *LAG3* expression analysis from the CheckMate 069 cohort (n=52 patients). **(A)** UMAP visualization of 30 identified cell populations from the CheckMate 069 scRNAseq cohort (n=100 samples). **(B)** Frequency of *LAG3* transcript expression in each of the identified cell populations. **(C)** Top differentially expressed genes between *LAG3^+^* and *LAG3^-^* cells from CD8 TCM cells (top) and TEM cells (bottom). **(D)** Expression of selected genes in *LAG3* ^+^ CD8 TCM cells (top) and TEM cells (bottom). Dot size corresponds to the frequency of the indicated cell population. Blue gradient bars display the average z-score normalized transcript expression level of the indicated gene in each respective cell population.

Finally, we sought to understand the potential relationship of LAG-3-expressing cells to the clinical outcomes of patients treated with immune checkpoint inhibitors (ICI). Peripheral, blood-derived samples are more accessible than tumor tissues, so we utilized single cell RNA-sequencing (scRNAseq) of PBMC samples as a highly sensitive orthogonal technique to identify and characterize rare circulating immune cells that express LAG-3. Sequencing and analyses were conducted on paired baseline (cycle 1, day 1; C1D1) and on-treatment (cycle 3, day 1; C3D1) blood samples from 52 melanoma patients treated with ICI in the phase 2 CheckMate 069 trial (29, 30). As visualized in **Figure 7A**, scRNAseq profiling identified 30 different cell types that included 7 different T-cell subsets. Consistent with the results of our TIL profiling by flow and mass cytometry, we observed the majority of *LAG3* transcript expression in CD8 TCM and TEM cells (10.7% and 14.4.%, respectively) and a minority expressed in hematopoietic stem cells (HSC, 7.8%) and NK cells (7.0%; **Figure 7B**). Comparison of the top differentially expressed genes between *LAG3* ^+^ and *LAG3* ^-^ CD8 memory T cells revealed increased expression of cytotoxicity genes (*GZMA*, *GZMB*, *GZMH*, *GZMK*, and *NKG7*) and specific cytokines/chemokines (*IL32* and *CCL5*) in the *LAG3* ^+^ cells (**Figure 7C**). Co-expression of *TIGIT* (13.4% in TCM, 11.4% in TEM) and *TOX* (12.9% in TCM, 16.2% in TEM), and to a lesser extent *PDCD1* (encoding PD-1; 4% in TCM, 5.8% in TEM), *HAVCR2* (encoding TIM-3; 4.1% in TCM, 3.8% in TEM), and *TNFRSF9* (encoding 4-1BB; 3.0% in TCM, 2.6% in TEM), was also observed in *LAG3* ^+^ CD8 memory T cells (**Figure 7D**).

**Figure 8:**
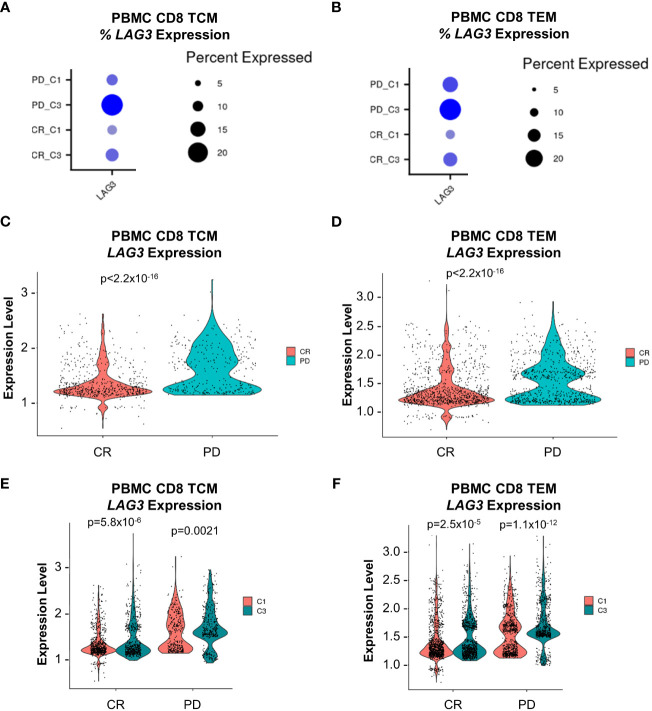
*LAG3* transcript expression analysis from peripheral CD8 TCM and TEM cells at baseline and on-treatment from the nivolumab + ipilimumab arm of the CheckMate 069 cohort (n=26 patients). **(A)** Frequency of *LAG3* transcript expression from CD8 TCM cells. **(B)** Frequency of *LAG3* transcript expression from CD8 TEM cells. **(C)** Comparison of the magnitude of *LAG3* transcript expression from *LAG3* ^+^ CD8 TCM cells at baseline (C1D1) between subjects with CR vs. PD. **(D)** Comparison of the magnitude of *LAG3* transcript expression from *LAG3* ^+^ CD8 TEM cells at baseline (C1D1) between subjects with CR vs. PD. **(E)** Comparison of the magnitude of *LAG3* transcript expression from *LAG3* ^+^ CD8 TCM cells at baseline (C1D1) and on-treatment (C3D1) between subjects with CR and PD. **(F)** *LAG3* transcript expression from *LAG3* ^+^ CD8 TEM cells at baseline (C1D1) and on-treatment (C3D1) between subjects with CR and PD. Dot size corresponds to the frequency of the indicated cell population and color corresponds to average expression. Violin plots display z-score normalized *LAG3* transcript expression in each respective patient population. C1D1, cycle 1 day 1; C3D1, cycle 3 day 1; CR, complete response; PD, progressive disease; SD, stable disease.

To investigate potential associations between *LAG3*-expressing CD8 memory T cells and clinical outcomes, we focused on 23 patients that were treated with combination nivolumab and ipilimumab whose disease was classified as complete response (CR, n=15) or progressive disease (PD, n=8). We observed an elevated baseline median frequency of circulating *LAG3* ^+^ CD8 TEM cells in patients who exhibited PD compared to CR (18.1% vs. 11.1%, respectively), but not for *LAG3* ^+^ CD8 TCM cells (10.7% vs. 9.1%, respectively; **Figures 8A, B**). Furthermore, the level of baseline *LAG3* transcript expression in TCM and TEM cells was significantly higher in patients with PD compared to CR (**Figures 8C, D**). We also observed an on-treatment increase in the median frequency of circulating *LAG3* ^+^ CD8 TCM and TEM cells in all patients at the cycle 3 timepoint. However, the median frequency was even more elevated in patients with PD compared to CR for both *LAG3* ^+^ CD8 TCM cells (21.5% vs. 12.5%) and TEM cells (24.7% vs. 16.5%; **Figures 8A, B**). Similarly, patients with PD showed a greater on-treatment increase in the level of *LAG3* transcript expression in circulating *LAG3* ^+^ CD8 TCM and TEM cells compared to those with CR (**Figures 8E, F**). These results suggest that a relatively higher level of *LAG3* ^+^ CD8 memory T cells at baseline, and a larger increase on-treatment, may be associated with poorer outcomes in melanoma patients treated with combination ICI.

## 4 Discussion

The use of comprehensive high-parameter immunophenotyping to characterize peripheral and tumor-infiltrating immune cells can yield translational insights that aid the development of IO therapies for solid tumors. In the present study, we utilized both mass and fluorescence cytometry to profile immune cell populations that express LAG-3 from two separate commercial cohorts from a diversity of solid tumor types. As expected, we found that the majority of LAG-3 was expressed by tumor-infiltrating, but not circulating, CD8 memory T cells, with lower, but detectable, levels of LAG-3 on tumor-infiltrating CD4 T-helper cells and Tregs. These results are supported by findings reported by others in melanoma and hepatocellular carcinoma (20, 44, 45). Others have also reported LAG-3 expression on human plasmacytoid DCs (46) and NK cells (47), but we did not observe appreciable expression of LAG-3 in any of these cell types which may reflect the relative paucity of such cells among TIL. These results indicate the importance of tumor-infiltrating CD8 memory and CD4 T cells as potential targets of therapeutic LAG-3 blockade.

In seeking to characterize the marker profile of the LAG-3-expressing cells, we utilized both unsupervised analysis and manual gating. These complimentary approaches determined that CD8 memory T cells that express LAG-3 exhibit a unique marker profile, with robust expression of multiple co-inhibitory receptors (PD-1, CTLA-4, TIGIT, and TIM-3), co-stimulatory receptors (4-1BB and ICOS), and activation markers (CD69 and HLA-DR). This is highlighted by the similar expression profiles of specific FlowSOM-derived metaclusters and the manually gated PD-1^+^ LAG-3^+^ subsets of CD8 memory T cells. Although the absolute number of TIL samples profiled across both cohorts here was somewhat limited (total n=45), this marker co-expression profile was generally consistent across both cohorts and was consistent with other reports focused on NSCLC and melanoma (16, 17). Taken together, this indicates that the association between LAG-3 expression and this distinct surface marker phenotype may be a general feature of solid tumor biology. Additionally, analysis of the tumor-infiltrating T cell tSNE maps revealed a much more localized and focal pattern of expression for LAG-3 (particularly surface LAG-3) compared to the other co-inhibitory and co-stimulatory receptors, which were more broadly expressed. It is tempting to speculate that expression profiling of LAG-3, together with PD-1, may be a simple way to enrich for tumor-infiltrating T cells that highly co-express multiple checkpoints and co-stimulators, as has been reported for PD-1^high^ CD8 T cells from NSCLC (18, 48) and ovarian cancer (38). Since we found that PD-1 and LAG-3 were frequently co-expressed in CD8 memory T cells, and that PD-1 expression intensity was equivalent or higher in cells that also expressed LAG-3, it seems likely that the PD-1^high^ CD8 T cell subset identified in these prior investigations is comparable to the PD-1^+^ LAG-3^+^ CD8 memory T cell subsets reported here.

Persistent antigen stimulation and immunosuppressive signals in the TME result in the dysfunction of T cells (43, 49) akin to the exhaustion model that was first described in chronic viral infection (50, 51). A key feature that identifies exhausted/dysfunctional T cells is relatively high expression of TOX compared to TCF1 (40–42), which we observed in the high LAG-3-expressing CD8 T cell metaclusters from the flow cytometry cohort. Exhausted and dysfunctional CD8 T cells also exhibit chronic upregulation of multiple inhibitory checkpoints (19), which was reported in the PD-1^high^ CD8 T cell subsets (18, 38) and was also observed in the PD-1^+^ LAG-3^+^ CD8 memory T cell subsets described in our study. Dysfunction in tumor infiltrating CD8 T cells is also associated with marker characteristics indicative of tumor reactivity, including CD4/CD8 double positivity (24), co-expression of CD103 and CD39 (52), expression of CXCL13 (53), and upregulation of 4-1BB, which reflects recent antigen encounter (54) and was reported to aid in discriminating tumor-reactive CD8 T cells from bystander CD8 T cells in the TME (55). Elevated 4-1BB expression was observed in the PD-1^+^ LAG-3^+^ CD8 memory T cell subsets profiled here, and in the PD-1^high^ CD8 T cell subsets described previously (18, 38). A potential association between LAG-3 and tumor reactivity is also suggested by a murine study that reported co-expression of LAG-3 and 4-1BB was sufficient to identify tumor antigen-specific yet dysfunctional CD8 T cells from syngeneic tumors, and that combination treatment with anti-LAG-3 and anti-4-1BB antibodies resulted in potent inhibition of tumor growth (56). The unique marker phenotype of the tumor-infiltrating PD-1^+^ LAG-3^+^ CD8 memory T cell subsets characterized here indicates that this subset of cells may similarly possess tumor-reactive yet dysfunctional features and may be amenable to reinvigoration by combination PD-1 and LAG-3 blockade. Retrospective analyses for any potential association(s) between this subset and patient responsiveness to dual PD-1 and LAG-3 blockade could be undertaken to determine the exploratory biomarker potential of this subset (as was recently shown for PD-1^high^ CD8 T cells from NSCLC (48)) and complement experimental efforts to determine whether this subset is a primary mechanistic target of dual blockade. This notion is further supported by recent exploratory subgroup analyses from the RELATIVITY-047 trial, in which the overall response rate (ORR) for the nivolumab + relatlimab arm was greater in patients whose tumors expressed both PD-L1 ≥ 1% + LAG-3 ≥ 1% (52.7%) compared to both the intention-to-treat (ITT) population (43.1%) and the PD-L1 < 1% + LAG-3 < 1% population (30.7%) (57). Additionally, novel approaches to safely target 4-1BB co-stimulation to the tumor and circumvent previously observed severe liver toxicity (58) may represent an attractive IO combination strategy to further augment PD-1 and LAG-3 dual blockade.

We also characterized the expression of LAG-3 ligands by broad immunophenotyping of tumor-infiltrating lymphocyte and myeloid populations. We observed relatively high expression of MHC-II across most immune populations, and galectin-3 and LSECtin expression in a minority of cells among the myeloid cell lineages. FGL1 was largely not expressed, with only a minimal signal seen in DCs. The diversity of LAG-3 ligands coupled with their observed expression on various immune cells (often at higher levels than what was observed for PD-L1) suggest that theoretically there would be abundant opportunities for LAG-3 to engage at least one of its receptors within the TME and signal accordingly. Recent investigations have detailed apparent contradictions in the ligand-dependency of the LAG-3 inhibitory mechanism: whereas Maruhashi et al. (59) and Wang et al. (9) first reported a requirement for LAG-3 engagement with either stable peptide:MHC-II (pMHCII) complexes or FGL1, respectively, a subsequent study by Guy et al. (12) reported an inhibitory mechanism for LAG-3 that was seemingly entirely independent of ligand engagement. Concurrently, a follow-up study by Maruhashi et al. (60) presented data reiterating the requirement for stable pMHCII engagement but found that engagement with FGL1 was dispensable for LAG-3-dependent inhibition. Further investigation will be necessary to resolve these apparent contradictions and yield a more complete understanding of the relative importance of the various ligands in potentially mediating antitumor T-cell responses through LAG-3.

Peripheral blood is much more accessible than solid tumor tissue, which can be difficult to collect, process, and preserve, and may not be as practical for routine clinical biomarker testing and/or repeated longitudinal sampling. We therefore pivoted from TIL to peripheral blood samples to explore the potential contribution of the LAG-3-expressing cell subsets to clinical outcomes of patients treated with ICI. Because we found very low levels of peripheral LAG-3 expression by CyTOF, we utilized scRNAseq as a highly sensitive orthogonal technique to profile and identify these rare circulating immune cell populations as they traffic to and from the tumor. Using samples from the phase 2 CheckMate 069 trial, we found that CD8 TEM and TCM cells expressed the majority of *LAG3*, which was entirely consistent with our TIL profiling results, and that the level of circulating *LAG3*-expressing CD8 memory T cells at baseline and on-treatment was inversely related to clinical response to combination PD-1 and CTLA-4 blockade. The putative association described here is supported by results reported by Shen et al., which identified a pre-treatment peripheral LAG^+^ immunotype that was defined in part by a LAG-3^+^ CD8 T cell population and predicted poorer outcomes to ICI in patients with melanoma and urothelial carcinoma (61). Taken together, these results are consistent with LAG-3 playing a key role in PD-1 and/or CTLA-4 blockade resistance, and provides additional rationale for targeting LAG-3 (in combination with PD-1) as a strategy to overcome ICI resistance in melanoma and possibly other solid tumor types.

Limitations related to the cytometry portions of this study include the use of TIL samples from a range of solid tumor types and stages, which may have influenced the observed immune phenotypes. The TIL samples also underwent digestion, processing, and freeze/thaw procedures. Membrane proteins such as LAG-3 can be sensitive to enzymatic digestion, thereby altering detection of the target protein. *In situ* techniques such as immunohistochemistry (IHC) and multiplex-immunofluorescence (IF) could be used in the future to complement the observations obtained by this study. Another technical limitation is that the unsupervised analysis, by necessity, utilized equal random down-sampling to the sample with the lowest event count, which could theoretically increase the probability of excluding very low frequency cell populations. Additionally, the immunophenotyping presented here focused exclusively on immune cells. Further studies would be needed to ascertain the expression characteristics of LAG-3 and its ligands on non-immune cell types, such as stromal, vascular, and tumor cells. Finally, our findings are consistent with the notion that the addition of LAG-3 blockade to PD-1 blockade could enhance the activity of tumor-reactive yet dysfunctional CD8 memory T cells in the TME, but our findings do not preclude a potential role for combination blockade in also augmenting T cell priming in the context of cancer (62). Future efforts could assess patients treated with combination PD-1 and LAG-3 blockade compared to only PD-1 blockade for changes in TCR clonality post-treatment to address this possibility.

Study limitations related to the scRNAseq portion of this study include the potential for discrepancies between transcript and protein expression, as distinct modes of regulation may independently control the expression levels of each type of molecule. For example, ectodomain shedding of LAG-3 protein by ADAM10 and ADAM17 (44, 63) may result in the absence of surface protein expression in cells that otherwise abundantly express *LAG3* transcript. While we did not observe abundant *PDCD1* transcript co-expression from the *LAG3*-expressing CD8 TCM and TEM cells, substantial co-expression of *TIGIT* and *TOX* transcripts was observed, suggesting that these circulating cells may be phenotypically similar to the populations that we described in the TIL compartment. In addition, the known scRNAseq “dropout effect” in which read-depth is limited resulting in failure to detect rarely expressed transcripts implies that the absence of detectable co-expression of a given transcript may not necessarily mean that there was in fact no expression in the target cell population. Our analyses of distinct datasets utilizing different methodologies converged on the significance of the LAG-3^+^ memory CD8 T cell populations, which highlights the potential importance of conducting further studies to scrutinize the dynamics of LAG-3 transcript vs. protein expression and regulation, and how these factors relate to CD8 T cell phenotype, function, and response to checkpoint inhibition.

In summary, we conducted a detailed pan-tumor expression profiling study of immune cells that express LAG-3 and its ligands. We found enrichment of LAG-3 protein expression in subsets of TIL CD8 memory T cells, which was associated with a unique phenotypic profile with elevated expression of multiple inhibitory and stimulatory markers. We also observed abundant expression of MHC-II, and variable expression of galectin-3 and LSECtin, among various TIL populations. Lastly, we observed in inverse relationship between baseline and on-treatment levels of circulating *LAG3*-expressing CD8 memory T cells and response to combination PD-1 and CTLA4 blockade in a clinical trial cohort of patients with melanoma. These results provide insights that can inform mechanistic hypotheses, treatment selection strategies, and novel combination therapy approaches to support continued development of dual PD-1 and LAG-3 blockade.

## Data availability statement

The raw cytometry data supporting the conclusions of this article will be made available by the authors, without undue reservation. The scRNAseq dataset presented in this article is not readily available because of existing confidential proprietary information and limitations of clinical trial participant consent. Requests to access the dataset should be directed to Bristol Myers Squibb in accordance with instructions provided (https://www.bms.com/researchers-and-partners/independent-research.html).

## Ethics statement

The studies involving humans were approved by qualified Independent Ethics Committees/Institutional Review Boards (IEC/IRB) at each clinical trial site. Please see the BMS Bioethics Policy statement for more details (https://www.bms.com/about-us/responsibility/position-on-key-issues/bioethics-policy-statement.html). The studies were conducted in accordance with the local legislation and institutional requirements. The participants provided their written informed consent to participate in this study.

## Author contributions

All authors contributed to planning the study and designing the experiments. CJ, SDa, SK, PMe, MJ, and SR performed the experiments. BG, SK, PMe, PMu, SDo, AL, JK, and JD analyzed the data. BG and JD wrote the manuscript with input from all authors. LM, NM-O, SDo, AL, JK, and JD supervised the study. All authors contributed to the article and approved the submitted version.
