# Supplementary material for: Comprehensive immunophenotyping of solid tumor-infiltrating immune cells reveals the expression characteristics of LAG-3 and its ligands
Source: Front Immunol. 2023 Sep 19;14:1151748. doi: 10.3389/fimmu.2023.1151748 (PMC10546411; doi:10.3389/fimmu.2023.1151748)
Supplement: Supplementary file 1 [file Presentation_1.pptx]

## Slide 1
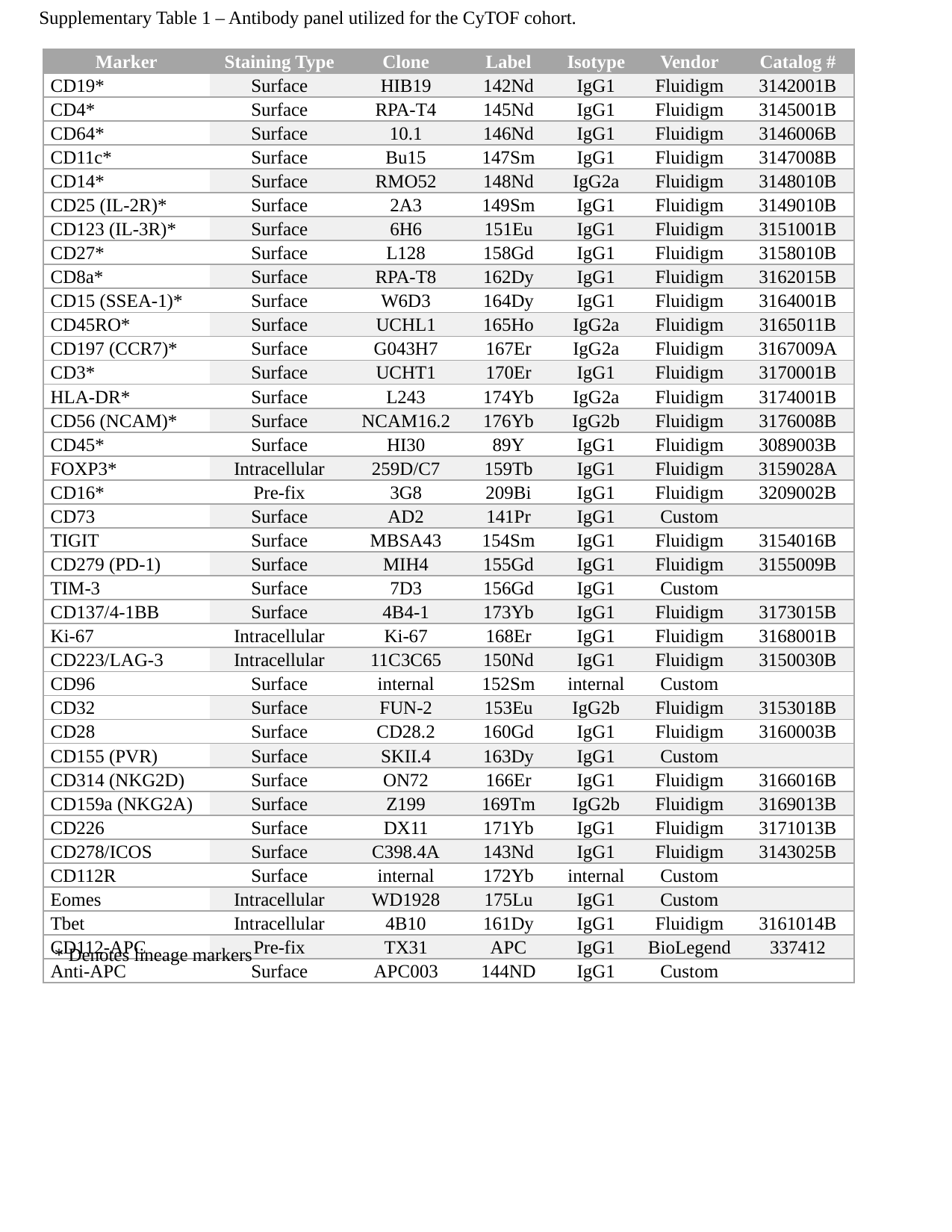

Supplementary Table 1 – Antibody panel utilized for the CyTOF cohort.
| Marker | Staining Type | Clone | Label | Isotype | Vendor | Catalog # |
| --- | --- | --- | --- | --- | --- | --- |
| CD19\* | Surface | HIB19 | 142Nd | IgG1 | Fluidigm | 3142001B |
| CD4\* | Surface | RPA-T4 | 145Nd | IgG1 | Fluidigm | 3145001B |
| CD64\* | Surface | 10.1 | 146Nd | IgG1 | Fluidigm | 3146006B |
| CD11c\* | Surface | Bu15 | 147Sm | IgG1 | Fluidigm | 3147008B |
| CD14\* | Surface | RMO52 | 148Nd | IgG2a | Fluidigm | 3148010B |
| CD25 (IL-2R)\* | Surface | 2A3 | 149Sm | IgG1 | Fluidigm | 3149010B |
| CD123 (IL-3R)\* | Surface | 6H6 | 151Eu | IgG1 | Fluidigm | 3151001B |
| CD27\* | Surface | L128 | 158Gd | IgG1 | Fluidigm | 3158010B |
| CD8a\* | Surface | RPA-T8 | 162Dy | IgG1 | Fluidigm | 3162015B |
| CD15 (SSEA-1)\* | Surface | W6D3 | 164Dy | IgG1 | Fluidigm | 3164001B |
| CD45RO\* | Surface | UCHL1 | 165Ho | IgG2a | Fluidigm | 3165011B |
| CD197 (CCR7)\* | Surface | G043H7 | 167Er | IgG2a | Fluidigm | 3167009A |
| CD3\* | Surface | UCHT1 | 170Er | IgG1 | Fluidigm | 3170001B |
| HLA-DR\* | Surface | L243 | 174Yb | IgG2a | Fluidigm | 3174001B |
| CD56 (NCAM)\* | Surface | NCAM16.2 | 176Yb | IgG2b | Fluidigm | 3176008B |
| CD45\* | Surface | HI30 | 89Y | IgG1 | Fluidigm | 3089003B |
| FOXP3\* | Intracellular | 259D/C7 | 159Tb | IgG1 | Fluidigm | 3159028A |
| CD16\* | Pre-fix | 3G8 | 209Bi | IgG1 | Fluidigm | 3209002B |
| CD73 | Surface | AD2 | 141Pr | IgG1 | Custom | |
| TIGIT | Surface | MBSA43 | 154Sm | IgG1 | Fluidigm | 3154016B |
| CD279 (PD-1) | Surface | MIH4 | 155Gd | IgG1 | Fluidigm | 3155009B |
| TIM-3 | Surface | 7D3 | 156Gd | IgG1 | Custom | |
| CD137/4-1BB | Surface | 4B4-1 | 173Yb | IgG1 | Fluidigm | 3173015B |
| Ki-67 | Intracellular | Ki-67 | 168Er | IgG1 | Fluidigm | 3168001B |
| CD223/LAG-3 | Intracellular | 11C3C65 | 150Nd | IgG1 | Fluidigm | 3150030B |
| CD96 | Surface | internal | 152Sm | internal | Custom | |
| CD32 | Surface | FUN-2 | 153Eu | IgG2b | Fluidigm | 3153018B |
| CD28 | Surface | CD28.2 | 160Gd | IgG1 | Fluidigm | 3160003B |
| CD155 (PVR) | Surface | SKII.4 | 163Dy | IgG1 | Custom | |
| CD314 (NKG2D) | Surface | ON72 | 166Er | IgG1 | Fluidigm | 3166016B |
| CD159a (NKG2A) | Surface | Z199 | 169Tm | IgG2b | Fluidigm | 3169013B |
| CD226 | Surface | DX11 | 171Yb | IgG1 | Fluidigm | 3171013B |
| CD278/ICOS | Surface | C398.4A | 143Nd | IgG1 | Fluidigm | 3143025B |
| CD112R | Surface | internal | 172Yb | internal | Custom | |
| Eomes | Intracellular | WD1928 | 175Lu | IgG1 | Custom | |
| Tbet | Intracellular | 4B10 | 161Dy | IgG1 | Fluidigm | 3161014B |
| CD112-APC | Pre-fix | TX31 | APC | IgG1 | BioLegend | 337412 |
| Anti-APC | Surface | APC003 | 144ND | IgG1 | Custom | |
* Denotes lineage markers

## Slide 2
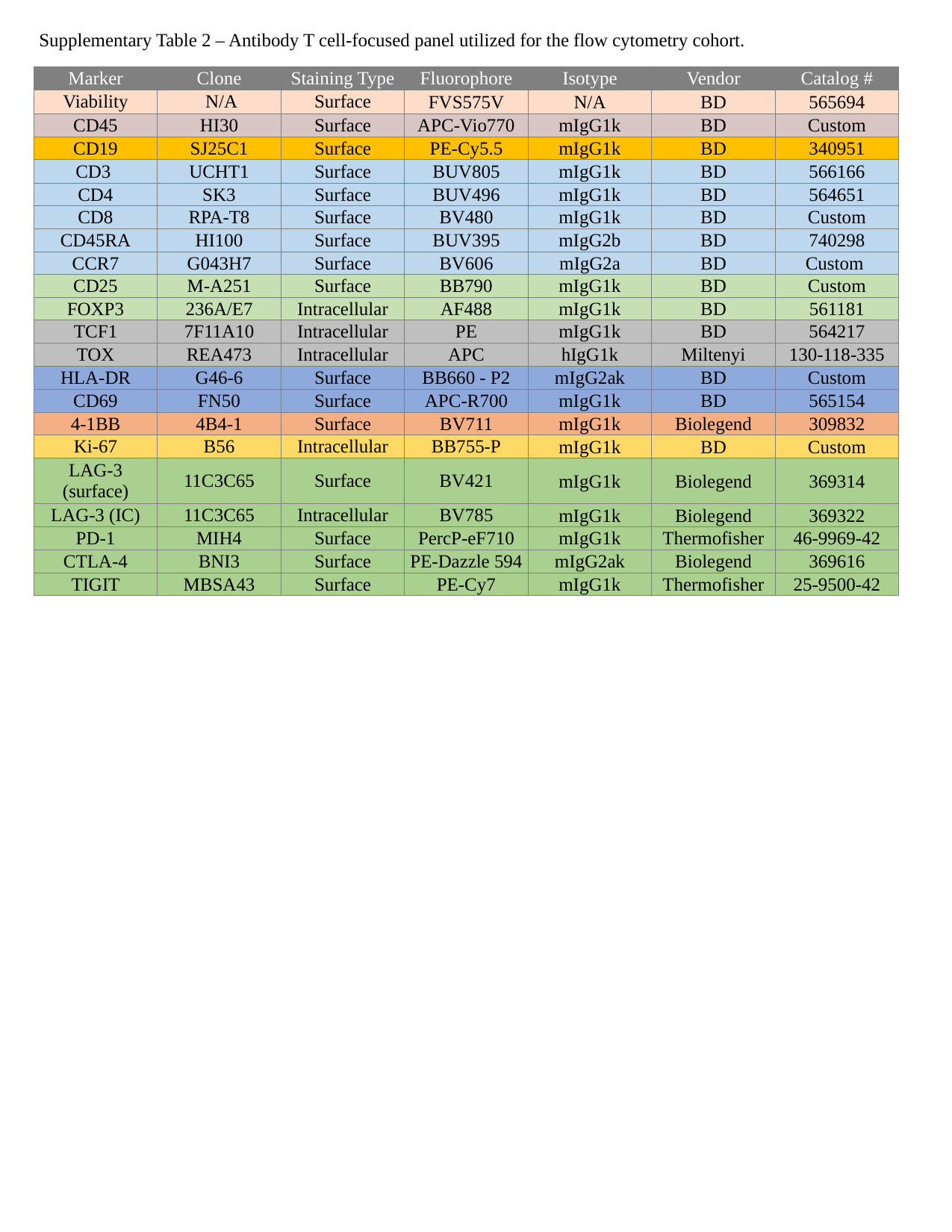

Supplementary Table 2 – Antibody T cell-focused panel utilized for the flow cytometry cohort.
| Marker | Clone | Staining Type | Fluorophore | Isotype | Vendor | Catalog # |
| --- | --- | --- | --- | --- | --- | --- |
| Viability | N/A | Surface | FVS575V | N/A | BD | 565694 |
| CD45 | HI30 | Surface | APC-Vio770 | mIgG1k | BD | Custom |
| CD19 | SJ25C1 | Surface | PE-Cy5.5 | mIgG1k | BD | 340951 |
| CD3 | UCHT1 | Surface | BUV805 | mIgG1k | BD | 566166 |
| CD4 | SK3 | Surface | BUV496 | mIgG1k | BD | 564651 |
| CD8 | RPA-T8 | Surface | BV480 | mIgG1k | BD | Custom |
| CD45RA | HI100 | Surface | BUV395 | mIgG2b | BD | 740298 |
| CCR7 | G043H7 | Surface | BV606 | mIgG2a | BD | Custom |
| CD25 | M-A251 | Surface | BB790 | mIgG1k | BD | Custom |
| FOXP3 | 236A/E7 | Intracellular | AF488 | mIgG1k | BD | 561181 |
| TCF1 | 7F11A10 | Intracellular | PE | mIgG1k | BD | 564217 |
| TOX | REA473 | Intracellular | APC | hIgG1k | Miltenyi | 130-118-335 |
| HLA-DR | G46-6 | Surface | BB660 - P2 | mIgG2ak | BD | Custom |
| CD69 | FN50 | Surface | APC-R700 | mIgG1k | BD | 565154 |
| 4-1BB | 4B4-1 | Surface | BV711 | mIgG1k | Biolegend | 309832 |
| Ki-67 | B56 | Intracellular | BB755-P | mIgG1k | BD | Custom |
| LAG-3 (surface) | 11C3C65 | Surface | BV421 | mIgG1k | Biolegend | 369314 |
| LAG-3 (IC) | 11C3C65 | Intracellular | BV785 | mIgG1k | Biolegend | 369322 |
| PD-1 | MIH4 | Surface | PercP-eF710 | mIgG1k | Thermofisher | 46-9969-42 |
| CTLA-4 | BNI3 | Surface | PE-Dazzle 594 | mIgG2ak | Biolegend | 369616 |
| TIGIT | MBSA43 | Surface | PE-Cy7 | mIgG1k | Thermofisher | 25-9500-42 |

## Slide 3
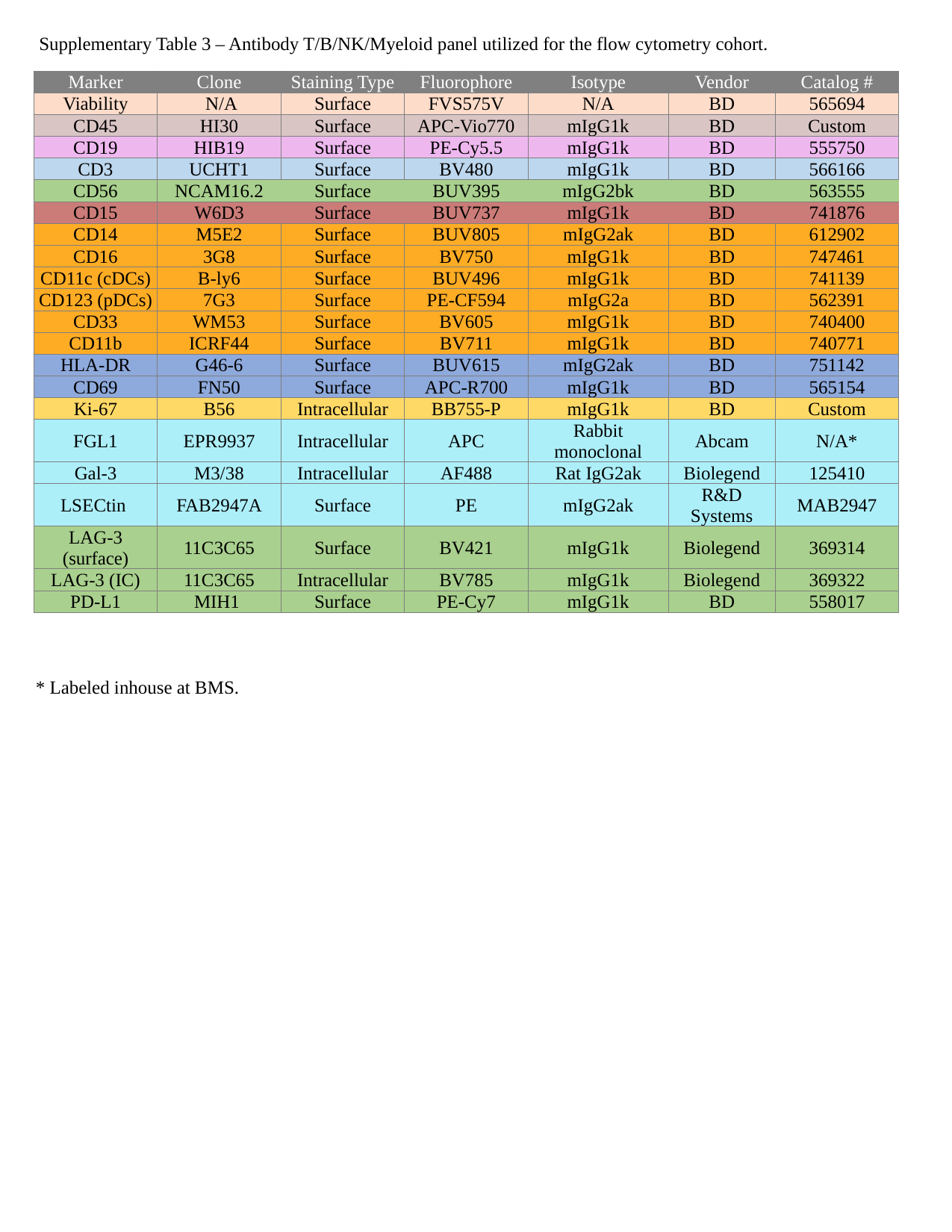

Supplementary Table 3 – Antibody T/B/NK/Myeloid panel utilized for the flow cytometry cohort.
| Marker | Clone | Staining Type | Fluorophore | Isotype | Vendor | Catalog # |
| --- | --- | --- | --- | --- | --- | --- |
| Viability | N/A | Surface | FVS575V | N/A | BD | 565694 |
| CD45 | HI30 | Surface | APC-Vio770 | mIgG1k | BD | Custom |
| CD19 | HIB19 | Surface | PE-Cy5.5 | mIgG1k | BD | 555750 |
| CD3 | UCHT1 | Surface | BV480 | mIgG1k | BD | 566166 |
| CD56 | NCAM16.2 | Surface | BUV395 | mIgG2bk | BD | 563555 |
| CD15 | W6D3 | Surface | BUV737 | mIgG1k | BD | 741876 |
| CD14 | M5E2 | Surface | BUV805 | mIgG2ak | BD | 612902 |
| CD16 | 3G8 | Surface | BV750 | mIgG1k | BD | 747461 |
| CD11c (cDCs) | B-ly6 | Surface | BUV496 | mIgG1k | BD | 741139 |
| CD123 (pDCs) | 7G3 | Surface | PE-CF594 | mIgG2a | BD | 562391 |
| CD33 | WM53 | Surface | BV605 | mIgG1k | BD | 740400 |
| CD11b | ICRF44 | Surface | BV711 | mIgG1k | BD | 740771 |
| HLA-DR | G46-6 | Surface | BUV615 | mIgG2ak | BD | 751142 |
| CD69 | FN50 | Surface | APC-R700 | mIgG1k | BD | 565154 |
| Ki-67 | B56 | Intracellular | BB755-P | mIgG1k | BD | Custom |
| FGL1 | EPR9937 | Intracellular | APC | Rabbit monoclonal | Abcam | N/A\* |
| Gal-3 | M3/38 | Intracellular | AF488 | Rat IgG2ak | Biolegend | 125410 |
| LSECtin | FAB2947A | Surface | PE | mIgG2ak | R&D Systems | MAB2947 |
| LAG-3 (surface) | 11C3C65 | Surface | BV421 | mIgG1k | Biolegend | 369314 |
| LAG-3 (IC) | 11C3C65 | Intracellular | BV785 | mIgG1k | Biolegend | 369322 |
| PD-L1 | MIH1 | Surface | PE-Cy7 | mIgG1k | BD | 558017 |
* Labeled inhouse at BMS.

## Slide 4
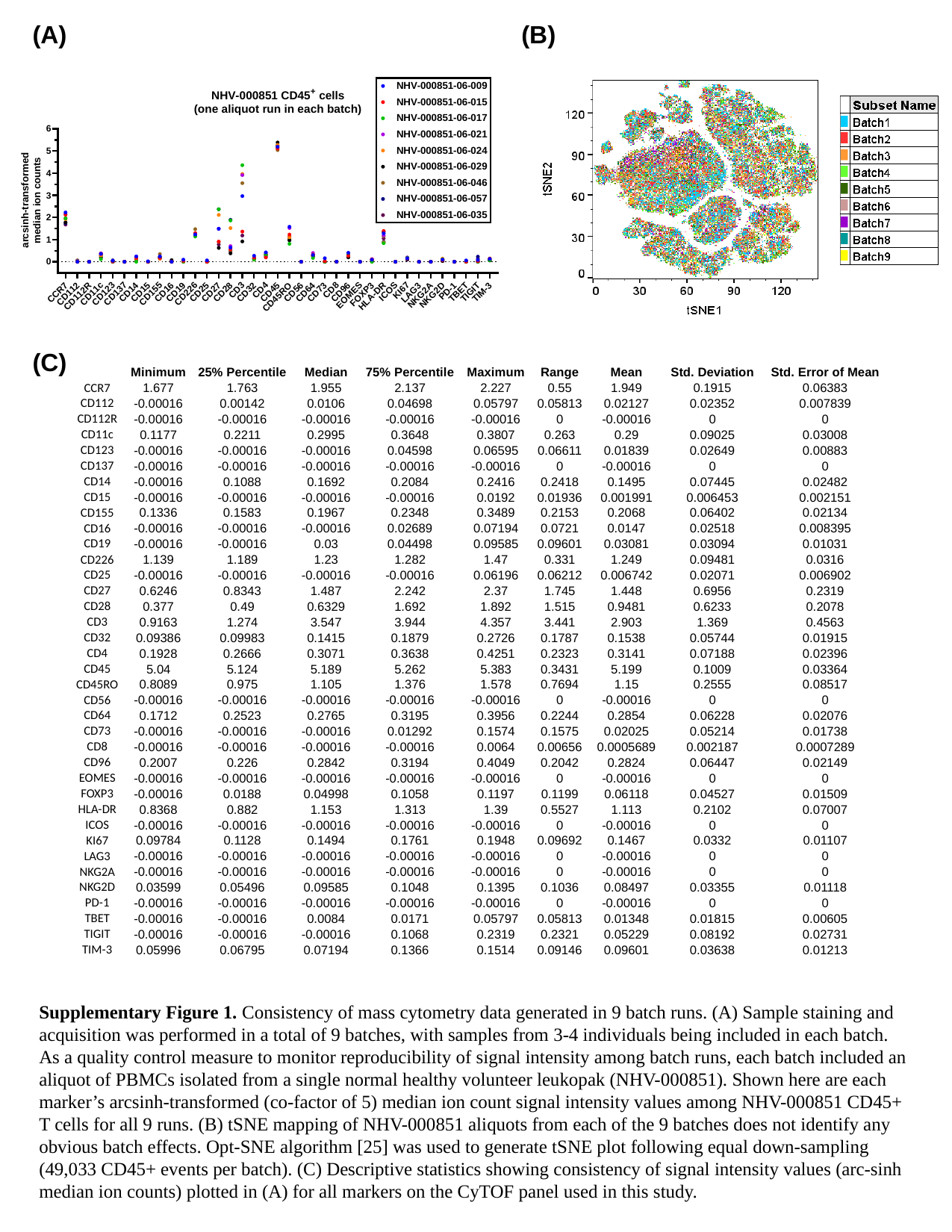

(A)
(B)
(C)
| | Minimum | 25% Percentile | Median | 75% Percentile | Maximum | Range | Mean | Std. Deviation | Std. Error of Mean |
| --- | --- | --- | --- | --- | --- | --- | --- | --- | --- |
| CCR7 | 1.677 | 1.763 | 1.955 | 2.137 | 2.227 | 0.55 | 1.949 | 0.1915 | 0.06383 |
| CD112 | -0.00016 | 0.00142 | 0.0106 | 0.04698 | 0.05797 | 0.05813 | 0.02127 | 0.02352 | 0.007839 |
| CD112R | -0.00016 | -0.00016 | -0.00016 | -0.00016 | -0.00016 | 0 | -0.00016 | 0 | 0 |
| CD11c | 0.1177 | 0.2211 | 0.2995 | 0.3648 | 0.3807 | 0.263 | 0.29 | 0.09025 | 0.03008 |
| CD123 | -0.00016 | -0.00016 | -0.00016 | 0.04598 | 0.06595 | 0.06611 | 0.01839 | 0.02649 | 0.00883 |
| CD137 | -0.00016 | -0.00016 | -0.00016 | -0.00016 | -0.00016 | 0 | -0.00016 | 0 | 0 |
| CD14 | -0.00016 | 0.1088 | 0.1692 | 0.2084 | 0.2416 | 0.2418 | 0.1495 | 0.07445 | 0.02482 |
| CD15 | -0.00016 | -0.00016 | -0.00016 | -0.00016 | 0.0192 | 0.01936 | 0.001991 | 0.006453 | 0.002151 |
| CD155 | 0.1336 | 0.1583 | 0.1967 | 0.2348 | 0.3489 | 0.2153 | 0.2068 | 0.06402 | 0.02134 |
| CD16 | -0.00016 | -0.00016 | -0.00016 | 0.02689 | 0.07194 | 0.0721 | 0.0147 | 0.02518 | 0.008395 |
| CD19 | -0.00016 | -0.00016 | 0.03 | 0.04498 | 0.09585 | 0.09601 | 0.03081 | 0.03094 | 0.01031 |
| CD226 | 1.139 | 1.189 | 1.23 | 1.282 | 1.47 | 0.331 | 1.249 | 0.09481 | 0.0316 |
| CD25 | -0.00016 | -0.00016 | -0.00016 | -0.00016 | 0.06196 | 0.06212 | 0.006742 | 0.02071 | 0.006902 |
| CD27 | 0.6246 | 0.8343 | 1.487 | 2.242 | 2.37 | 1.745 | 1.448 | 0.6956 | 0.2319 |
| CD28 | 0.377 | 0.49 | 0.6329 | 1.692 | 1.892 | 1.515 | 0.9481 | 0.6233 | 0.2078 |
| CD3 | 0.9163 | 1.274 | 3.547 | 3.944 | 4.357 | 3.441 | 2.903 | 1.369 | 0.4563 |
| CD32 | 0.09386 | 0.09983 | 0.1415 | 0.1879 | 0.2726 | 0.1787 | 0.1538 | 0.05744 | 0.01915 |
| CD4 | 0.1928 | 0.2666 | 0.3071 | 0.3638 | 0.4251 | 0.2323 | 0.3141 | 0.07188 | 0.02396 |
| CD45 | 5.04 | 5.124 | 5.189 | 5.262 | 5.383 | 0.3431 | 5.199 | 0.1009 | 0.03364 |
| CD45RO | 0.8089 | 0.975 | 1.105 | 1.376 | 1.578 | 0.7694 | 1.15 | 0.2555 | 0.08517 |
| CD56 | -0.00016 | -0.00016 | -0.00016 | -0.00016 | -0.00016 | 0 | -0.00016 | 0 | 0 |
| CD64 | 0.1712 | 0.2523 | 0.2765 | 0.3195 | 0.3956 | 0.2244 | 0.2854 | 0.06228 | 0.02076 |
| CD73 | -0.00016 | -0.00016 | -0.00016 | 0.01292 | 0.1574 | 0.1575 | 0.02025 | 0.05214 | 0.01738 |
| CD8 | -0.00016 | -0.00016 | -0.00016 | -0.00016 | 0.0064 | 0.00656 | 0.0005689 | 0.002187 | 0.0007289 |
| CD96 | 0.2007 | 0.226 | 0.2842 | 0.3194 | 0.4049 | 0.2042 | 0.2824 | 0.06447 | 0.02149 |
| EOMES | -0.00016 | -0.00016 | -0.00016 | -0.00016 | -0.00016 | 0 | -0.00016 | 0 | 0 |
| FOXP3 | -0.00016 | 0.0188 | 0.04998 | 0.1058 | 0.1197 | 0.1199 | 0.06118 | 0.04527 | 0.01509 |
| HLA-DR | 0.8368 | 0.882 | 1.153 | 1.313 | 1.39 | 0.5527 | 1.113 | 0.2102 | 0.07007 |
| ICOS | -0.00016 | -0.00016 | -0.00016 | -0.00016 | -0.00016 | 0 | -0.00016 | 0 | 0 |
| KI67 | 0.09784 | 0.1128 | 0.1494 | 0.1761 | 0.1948 | 0.09692 | 0.1467 | 0.0332 | 0.01107 |
| LAG3 | -0.00016 | -0.00016 | -0.00016 | -0.00016 | -0.00016 | 0 | -0.00016 | 0 | 0 |
| NKG2A | -0.00016 | -0.00016 | -0.00016 | -0.00016 | -0.00016 | 0 | -0.00016 | 0 | 0 |
| NKG2D | 0.03599 | 0.05496 | 0.09585 | 0.1048 | 0.1395 | 0.1036 | 0.08497 | 0.03355 | 0.01118 |
| PD-1 | -0.00016 | -0.00016 | -0.00016 | -0.00016 | -0.00016 | 0 | -0.00016 | 0 | 0 |
| TBET | -0.00016 | -0.00016 | 0.0084 | 0.0171 | 0.05797 | 0.05813 | 0.01348 | 0.01815 | 0.00605 |
| TIGIT | -0.00016 | -0.00016 | -0.00016 | 0.1068 | 0.2319 | 0.2321 | 0.05229 | 0.08192 | 0.02731 |
| TIM-3 | 0.05996 | 0.06795 | 0.07194 | 0.1366 | 0.1514 | 0.09146 | 0.09601 | 0.03638 | 0.01213 |
Supplementary Figure 1. Consistency of mass cytometry data generated in 9 batch runs. (A) Sample staining and acquisition was performed in a total of 9 batches, with samples from 3-4 individuals being included in each batch. As a quality control measure to monitor reproducibility of signal intensity among batch runs, each batch included an aliquot of PBMCs isolated from a single normal healthy volunteer leukopak (NHV-000851). Shown here are each marker’s arcsinh-transformed (co-factor of 5) median ion count signal intensity values among NHV-000851 CD45+ T cells for all 9 runs. (B) tSNE mapping of NHV-000851 aliquots from each of the 9 batches does not identify any obvious batch effects. Opt-SNE algorithm [25] was used to generate tSNE plot following equal down-sampling (49,033 CD45+ events per batch). (C) Descriptive statistics showing consistency of signal intensity values (arc-sinh median ion counts) plotted in (A) for all markers on the CyTOF panel used in this study.

## Slide 5
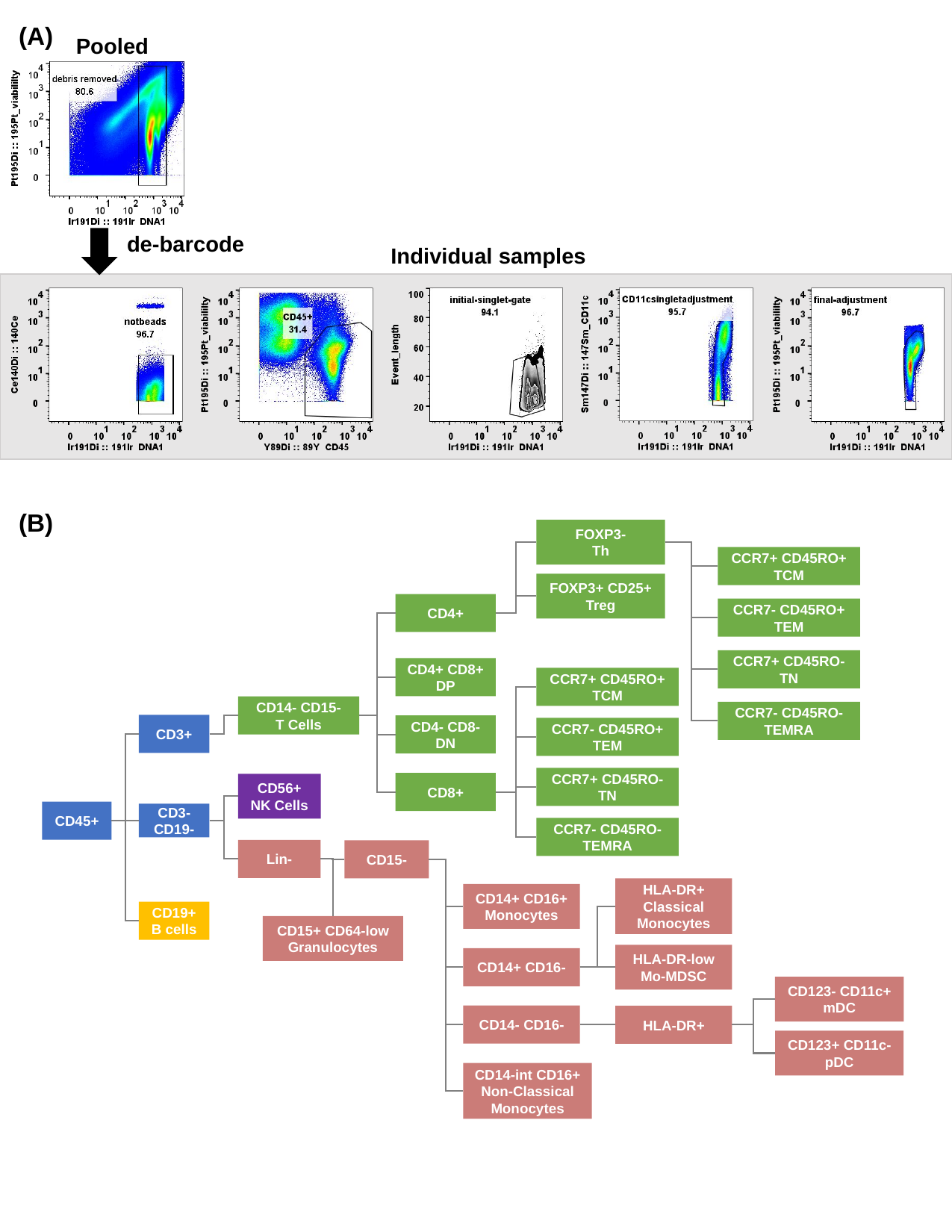

(A)
Pooled
de-barcode
Individual samples
(B)
FOXP3-
Th
CCR7+ CD45RO+
TCM
FOXP3+ CD25+
Treg
CD4+
CCR7- CD45RO+
TEM
CCR7+ CD45RO-
TN
CD4+ CD8+
DP
CCR7+ CD45RO+
TCM
CD14- CD15-
T Cells
CCR7- CD45RO-
TEMRA
CD3+
CD4- CD8-
DN
CCR7- CD45RO+
TEM
CCR7+ CD45RO-
TN
CD8+
CD56+
NK Cells
CD45+
CD3- CD19-
CCR7- CD45RO-
TEMRA
Lin-
CD15-
HLA-DR+
Classical Monocytes
CD14+ CD16+
Monocytes
CD19+
B cells
CD15+ CD64-low
Granulocytes
HLA-DR-low
Mo-MDSC
CD14+ CD16-
CD123- CD11c+
mDC
CD14- CD16-
HLA-DR+
CD123+ CD11c-
pDC
CD14-int CD16+
Non-Classical Monocytes

## Slide 6
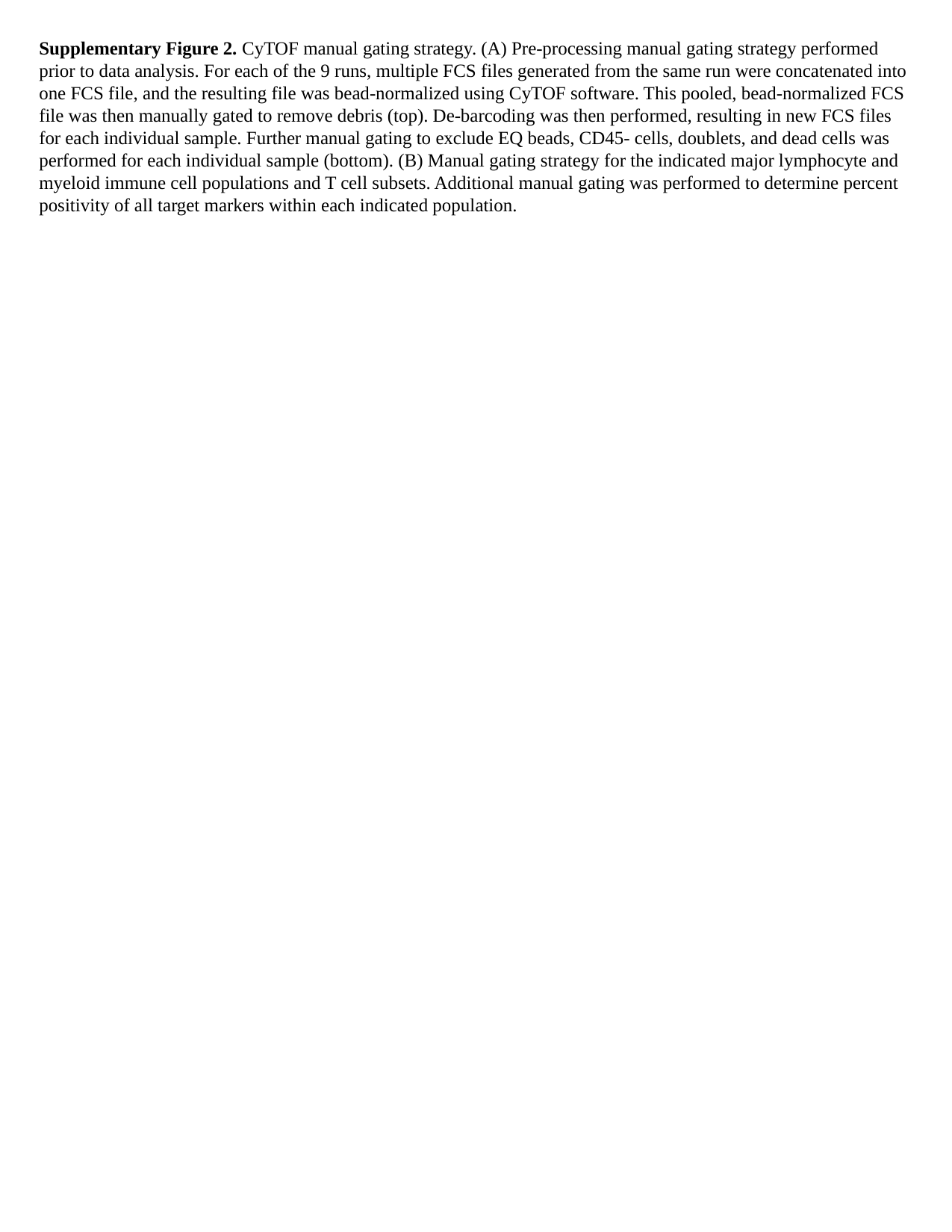

Supplementary Figure 2. CyTOF manual gating strategy. (A) Pre-processing manual gating strategy performed prior to data analysis. For each of the 9 runs, multiple FCS files generated from the same run were concatenated into one FCS file, and the resulting file was bead-normalized using CyTOF software. This pooled, bead-normalized FCS file was then manually gated to remove debris (top). De-barcoding was then performed, resulting in new FCS files for each individual sample. Further manual gating to exclude EQ beads, CD45- cells, doublets, and dead cells was performed for each individual sample (bottom). (B) Manual gating strategy for the indicated major lymphocyte and myeloid immune cell populations and T cell subsets. Additional manual gating was performed to determine percent positivity of all target markers within each indicated population.

## Slide 7
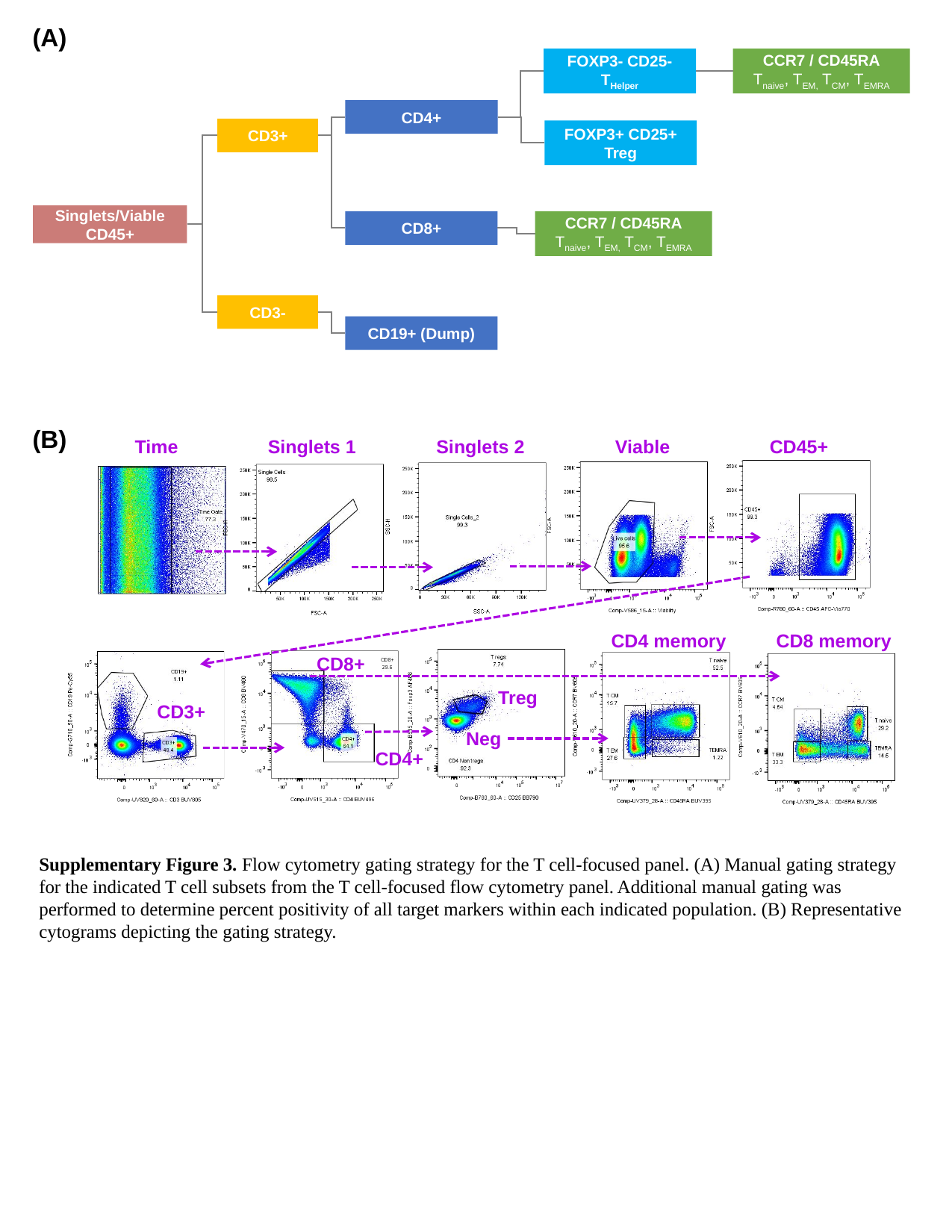

(A)
FOXP3- CD25-
THelper
CCR7 / CD45RA
Tnaive, TEM, TCM, TEMRA
CD4+
CD3+
FOXP3+ CD25+
Treg
Singlets/Viable
CD45+
CD8+
CCR7 / CD45RA
Tnaive, TEM, TCM, TEMRA
CD3-
CD19+ (Dump)
(B)
Time
Singlets 1
Singlets 2
Viable
CD45+
CD4 memory
CD8 memory
CD8+
Treg
CD3+
Neg
CD4+
Supplementary Figure 3. Flow cytometry gating strategy for the T cell-focused panel. (A) Manual gating strategy for the indicated T cell subsets from the T cell-focused flow cytometry panel. Additional manual gating was performed to determine percent positivity of all target markers within each indicated population. (B) Representative cytograms depicting the gating strategy.

## Slide 8
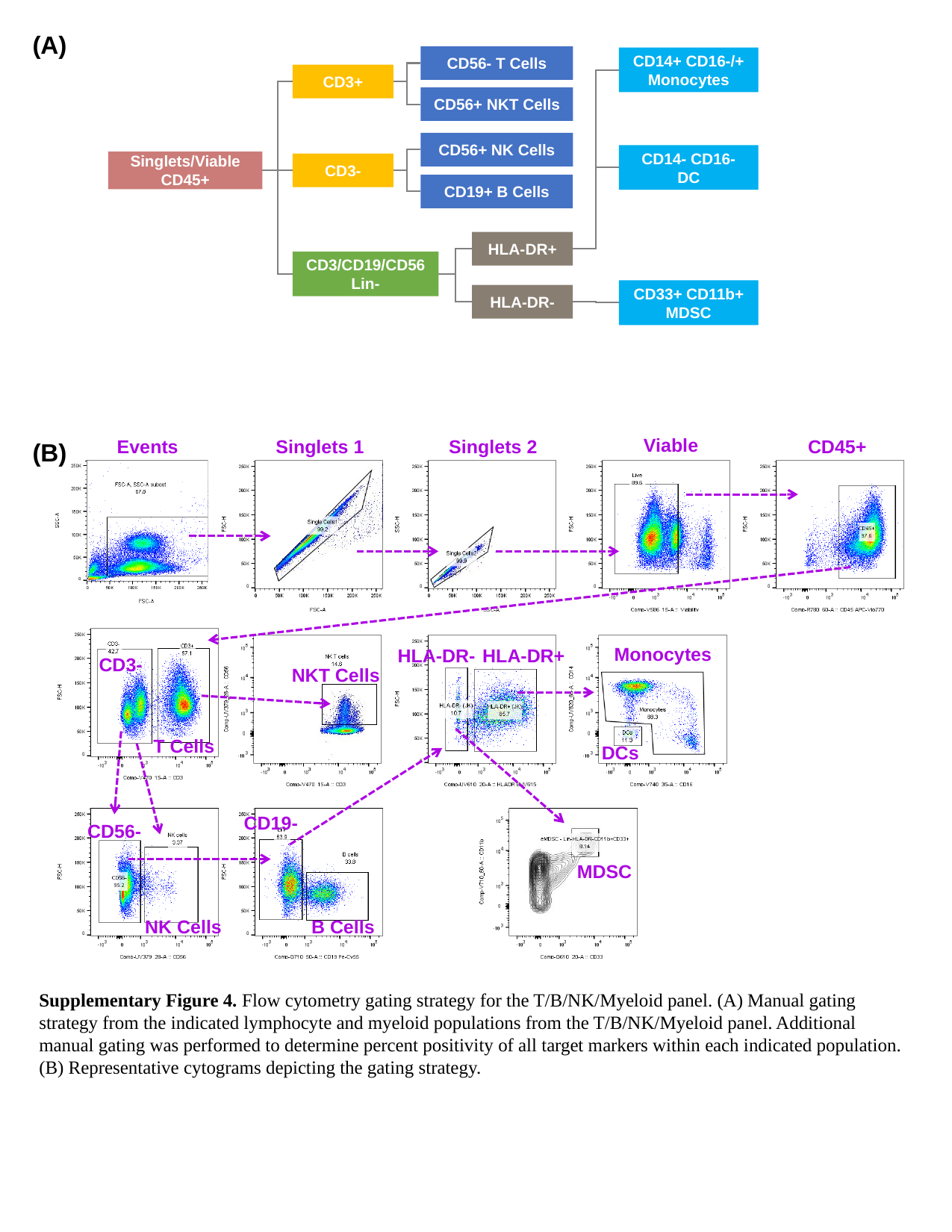

(A)
CD56- T Cells
CD14+ CD16-/+
Monocytes
CD3+
CD56+ NKT Cells
CD56+ NK Cells
CD14- CD16-
DC
Singlets/Viable
CD45+
CD3-
CD19+ B Cells
HLA-DR+
CD3/CD19/CD56
Lin-
CD33+ CD11b+
MDSC
HLA-DR-
Viable
Events
Singlets 1
Singlets 2
CD45+
(B)
Monocytes
HLA-DR-
HLA-DR+
CD3-
NKT Cells
T Cells
DCs
CD19-
CD56-
MDSC
B Cells
NK Cells
Supplementary Figure 4. Flow cytometry gating strategy for the T/B/NK/Myeloid panel. (A) Manual gating strategy from the indicated lymphocyte and myeloid populations from the T/B/NK/Myeloid panel. Additional manual gating was performed to determine percent positivity of all target markers within each indicated population. (B) Representative cytograms depicting the gating strategy.

## Slide 9
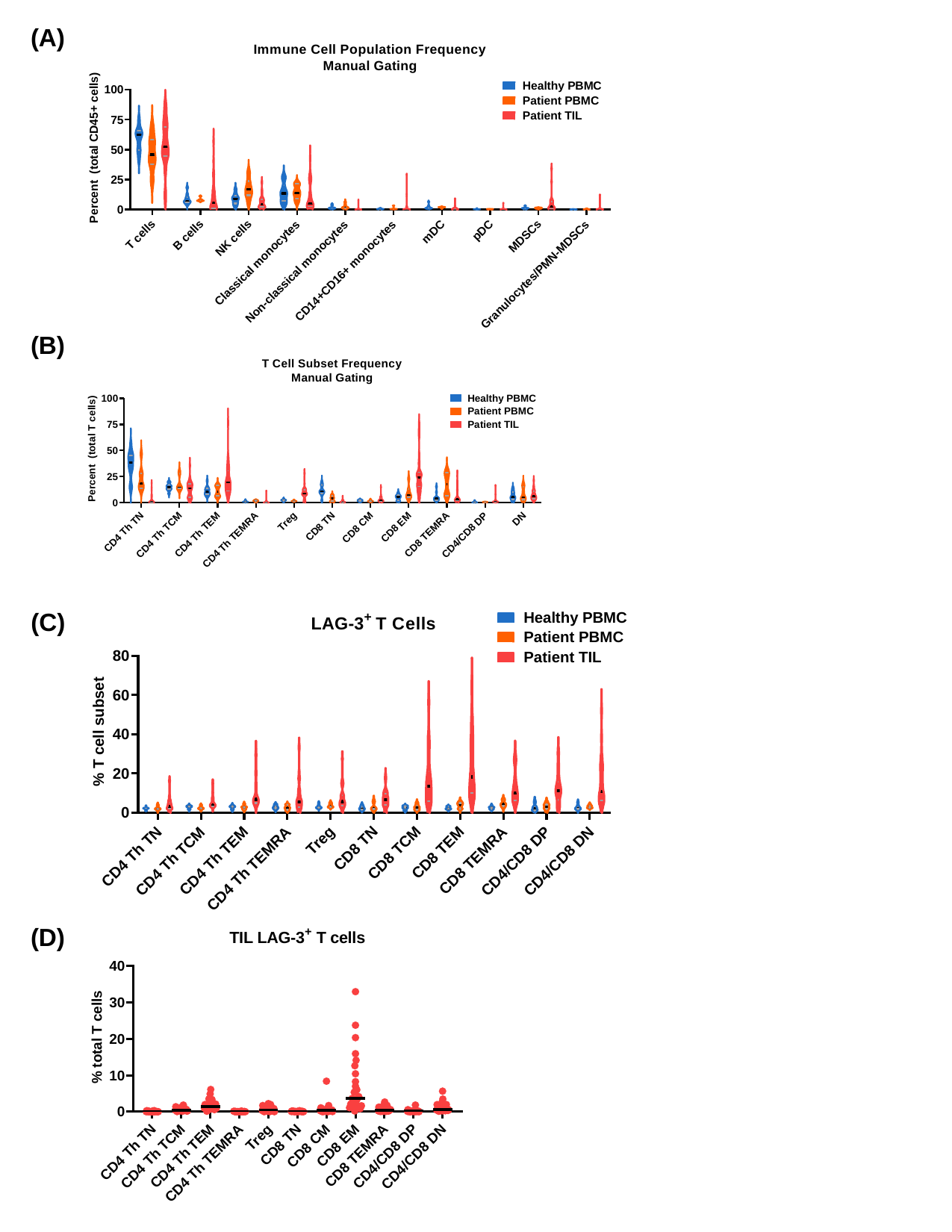

(A)
(B)
(C)
(D)

## Slide 10
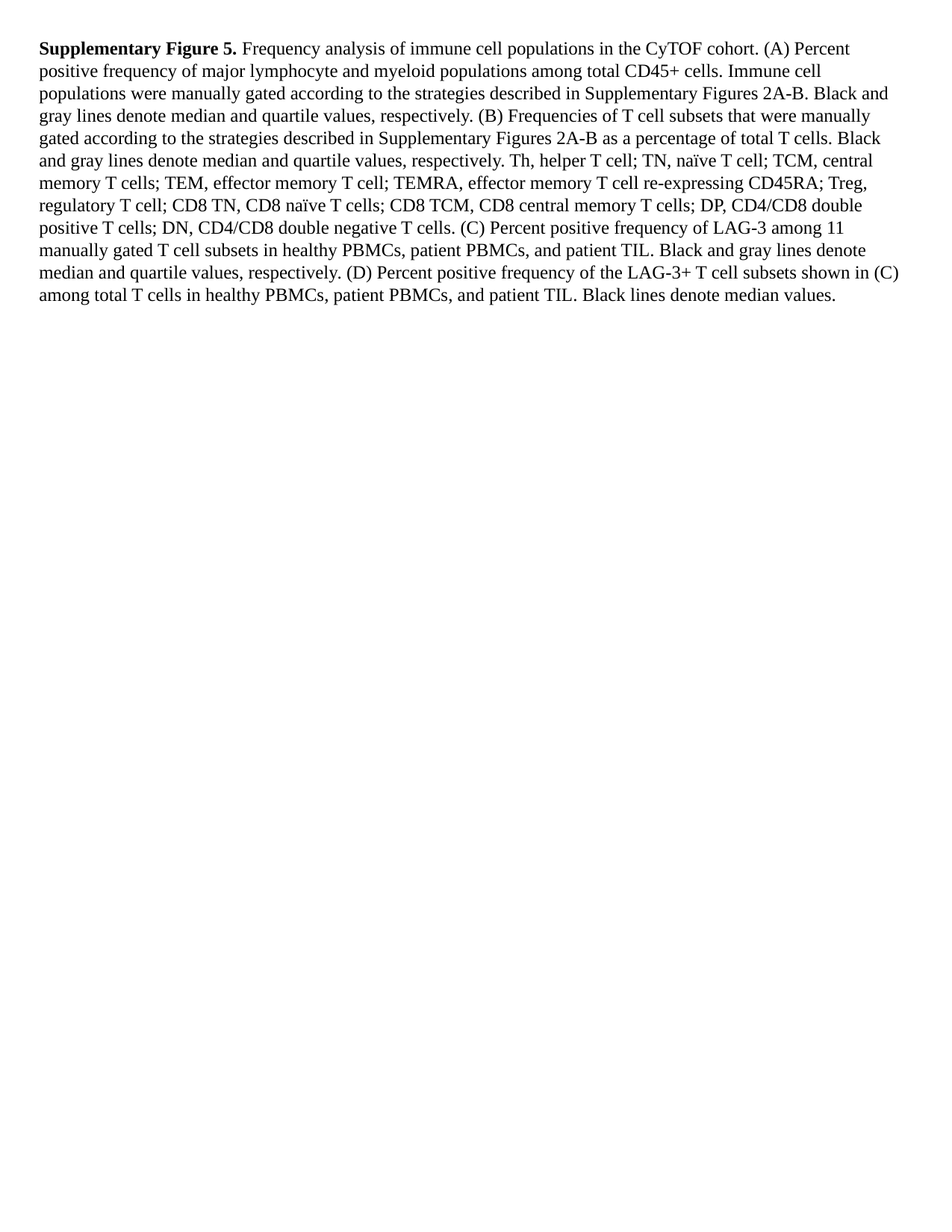

Supplementary Figure 5. Frequency analysis of immune cell populations in the CyTOF cohort. (A) Percent positive frequency of major lymphocyte and myeloid populations among total CD45+ cells. Immune cell populations were manually gated according to the strategies described in Supplementary Figures 2A-B. Black and gray lines denote median and quartile values, respectively. (B) Frequencies of T cell subsets that were manually gated according to the strategies described in Supplementary Figures 2A-B as a percentage of total T cells. Black and gray lines denote median and quartile values, respectively. Th, helper T cell; TN, naïve T cell; TCM, central memory T cells; TEM, effector memory T cell; TEMRA, effector memory T cell re-expressing CD45RA; Treg, regulatory T cell; CD8 TN, CD8 naïve T cells; CD8 TCM, CD8 central memory T cells; DP, CD4/CD8 double positive T cells; DN, CD4/CD8 double negative T cells. (C) Percent positive frequency of LAG-3 among 11 manually gated T cell subsets in healthy PBMCs, patient PBMCs, and patient TIL. Black and gray lines denote median and quartile values, respectively. (D) Percent positive frequency of the LAG-3+ T cell subsets shown in (C) among total T cells in healthy PBMCs, patient PBMCs, and patient TIL. Black lines denote median values.

## Slide 11
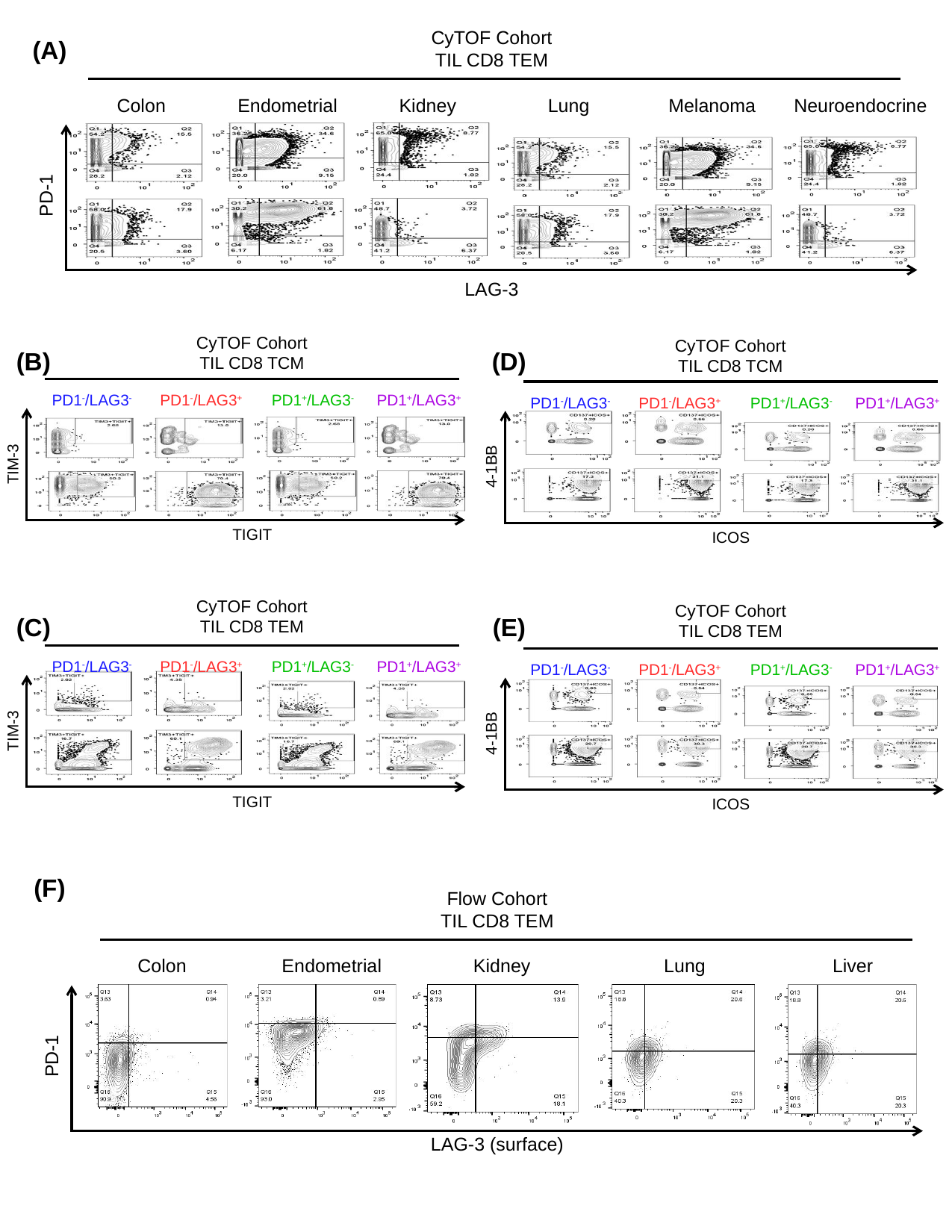

CyTOF Cohort
TIL CD8 TEM
(A)
Colon
Endometrial
Kidney
Lung
Melanoma
Neuroendocrine
PD-1
LAG-3
CyTOF Cohort
TIL CD8 TCM
CyTOF Cohort
TIL CD8 TCM
(B)
(D)
PD1-/LAG3-
PD1-/LAG3+
PD1+/LAG3-
PD1+/LAG3+
PD1-/LAG3-
PD1-/LAG3+
PD1+/LAG3-
PD1+/LAG3+
TIM-3
4-1BB
TIGIT
ICOS
CyTOF Cohort
TIL CD8 TEM
CyTOF Cohort
TIL CD8 TEM
(E)
(C)
PD1-/LAG3-
PD1-/LAG3+
PD1+/LAG3-
PD1+/LAG3+
PD1-/LAG3-
PD1-/LAG3+
PD1+/LAG3-
PD1+/LAG3+
TIM-3
4-1BB
TIGIT
ICOS
(F)
Flow Cohort
TIL CD8 TEM
Colon
Endometrial
Kidney
Lung
Liver
PD-1
LAG-3 (surface)

## Slide 12
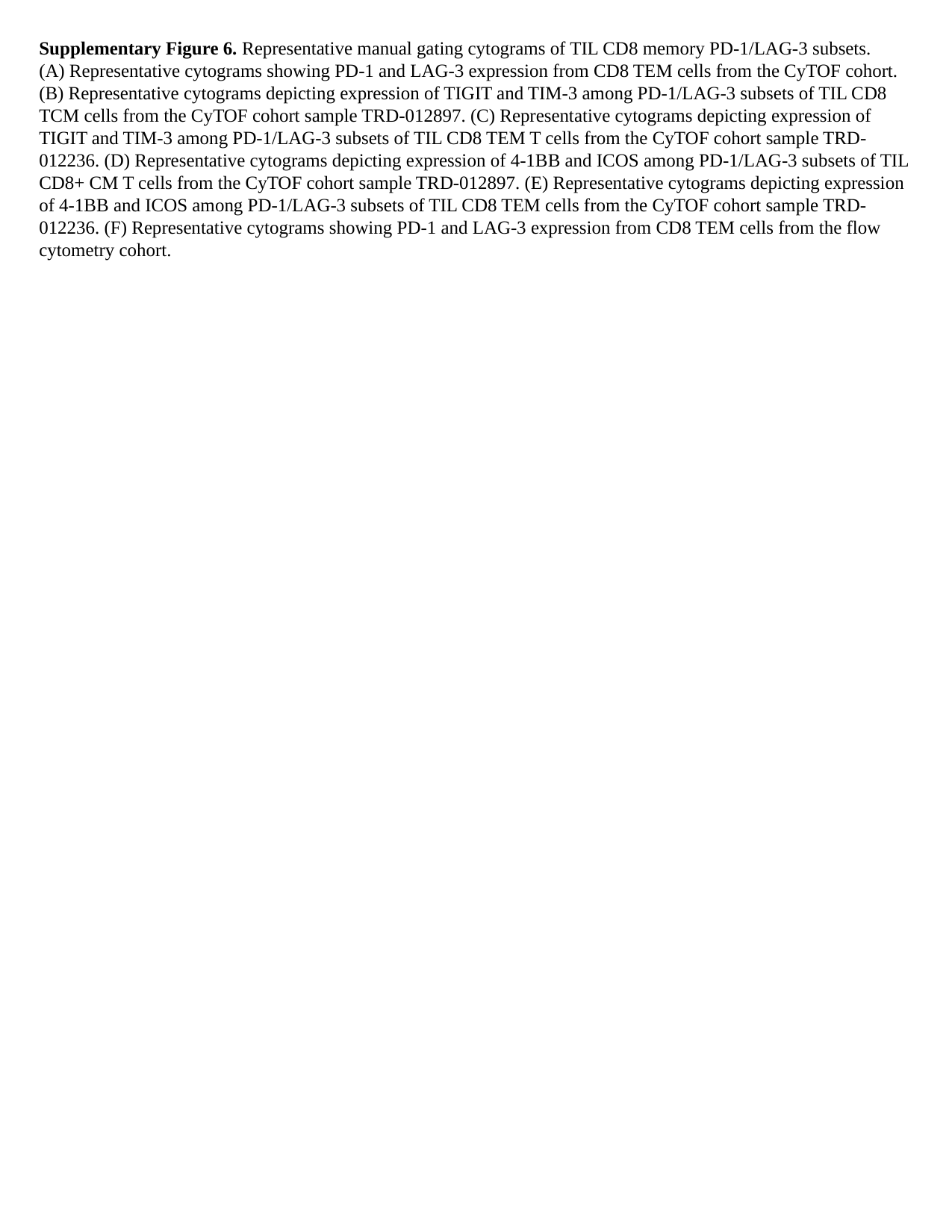

Supplementary Figure 6. Representative manual gating cytograms of TIL CD8 memory PD-1/LAG-3 subsets.
(A) Representative cytograms showing PD-1 and LAG-3 expression from CD8 TEM cells from the CyTOF cohort. (B) Representative cytograms depicting expression of TIGIT and TIM-3 among PD-1/LAG-3 subsets of TIL CD8 TCM cells from the CyTOF cohort sample TRD-012897. (C) Representative cytograms depicting expression of TIGIT and TIM-3 among PD-1/LAG-3 subsets of TIL CD8 TEM T cells from the CyTOF cohort sample TRD-012236. (D) Representative cytograms depicting expression of 4-1BB and ICOS among PD-1/LAG-3 subsets of TIL CD8+ CM T cells from the CyTOF cohort sample TRD-012897. (E) Representative cytograms depicting expression of 4-1BB and ICOS among PD-1/LAG-3 subsets of TIL CD8 TEM cells from the CyTOF cohort sample TRD-012236. (F) Representative cytograms showing PD-1 and LAG-3 expression from CD8 TEM cells from the flow cytometry cohort.

## Slide 13
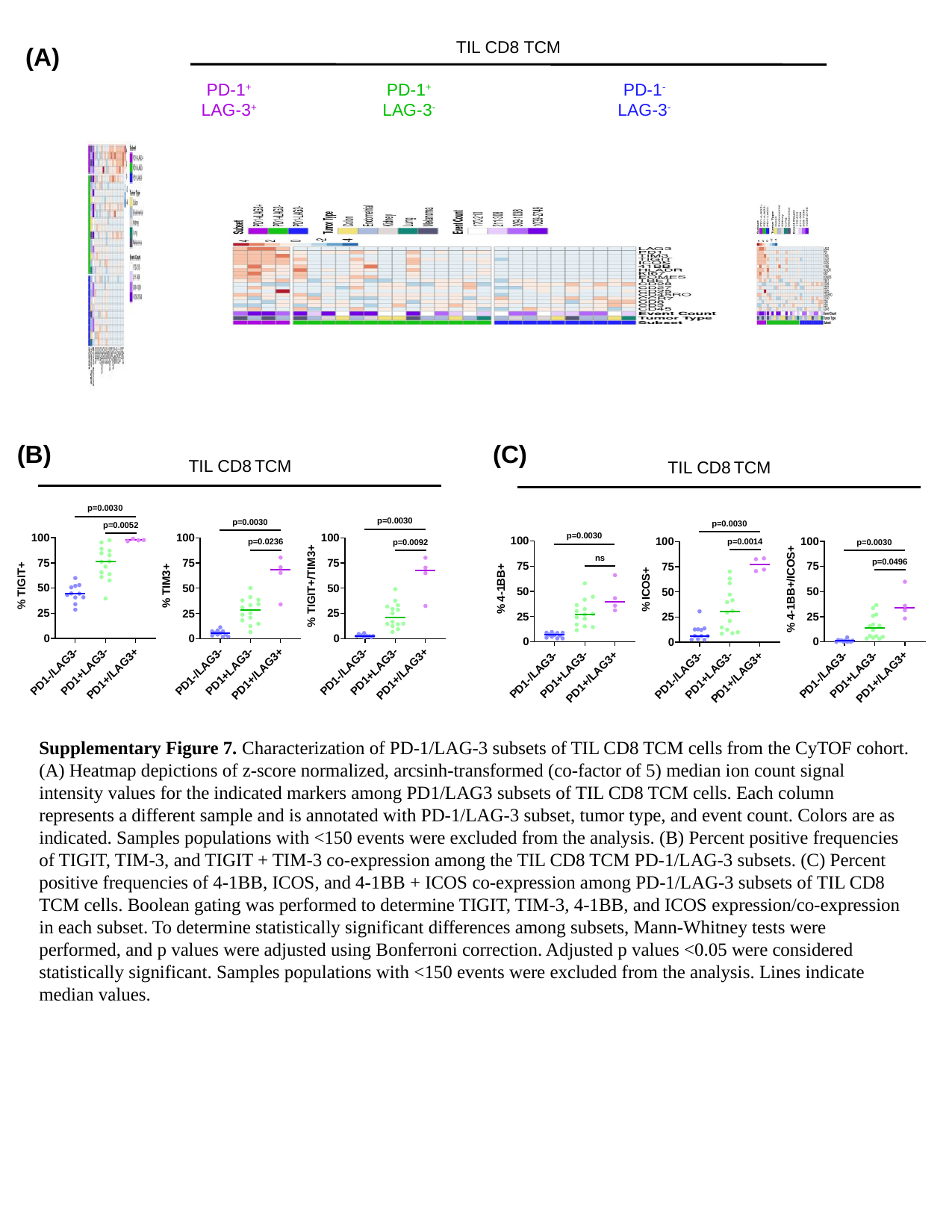

TIL CD8 TCM
PD-1+
LAG-3+
PD-1+
LAG-3-
PD-1-
LAG-3-
(A)
(B)
(C)
TIL CD8 TCM
TIL CD8 TCM
Supplementary Figure 7. Characterization of PD-1/LAG-3 subsets of TIL CD8 TCM cells from the CyTOF cohort.
(A) Heatmap depictions of z-score normalized, arcsinh-transformed (co-factor of 5) median ion count signal intensity values for the indicated markers among PD1/LAG3 subsets of TIL CD8 TCM cells. Each column represents a different sample and is annotated with PD-1/LAG-3 subset, tumor type, and event count. Colors are as indicated. Samples populations with <150 events were excluded from the analysis. (B) Percent positive frequencies of TIGIT, TIM-3, and TIGIT + TIM-3 co-expression among the TIL CD8 TCM PD-1/LAG-3 subsets. (C) Percent positive frequencies of 4-1BB, ICOS, and 4-1BB + ICOS co-expression among PD-1/LAG-3 subsets of TIL CD8 TCM cells. Boolean gating was performed to determine TIGIT, TIM-3, 4-1BB, and ICOS expression/co-expression in each subset. To determine statistically significant differences among subsets, Mann-Whitney tests were performed, and p values were adjusted using Bonferroni correction. Adjusted p values <0.05 were considered statistically significant. Samples populations with <150 events were excluded from the analysis. Lines indicate median values.

## Slide 14
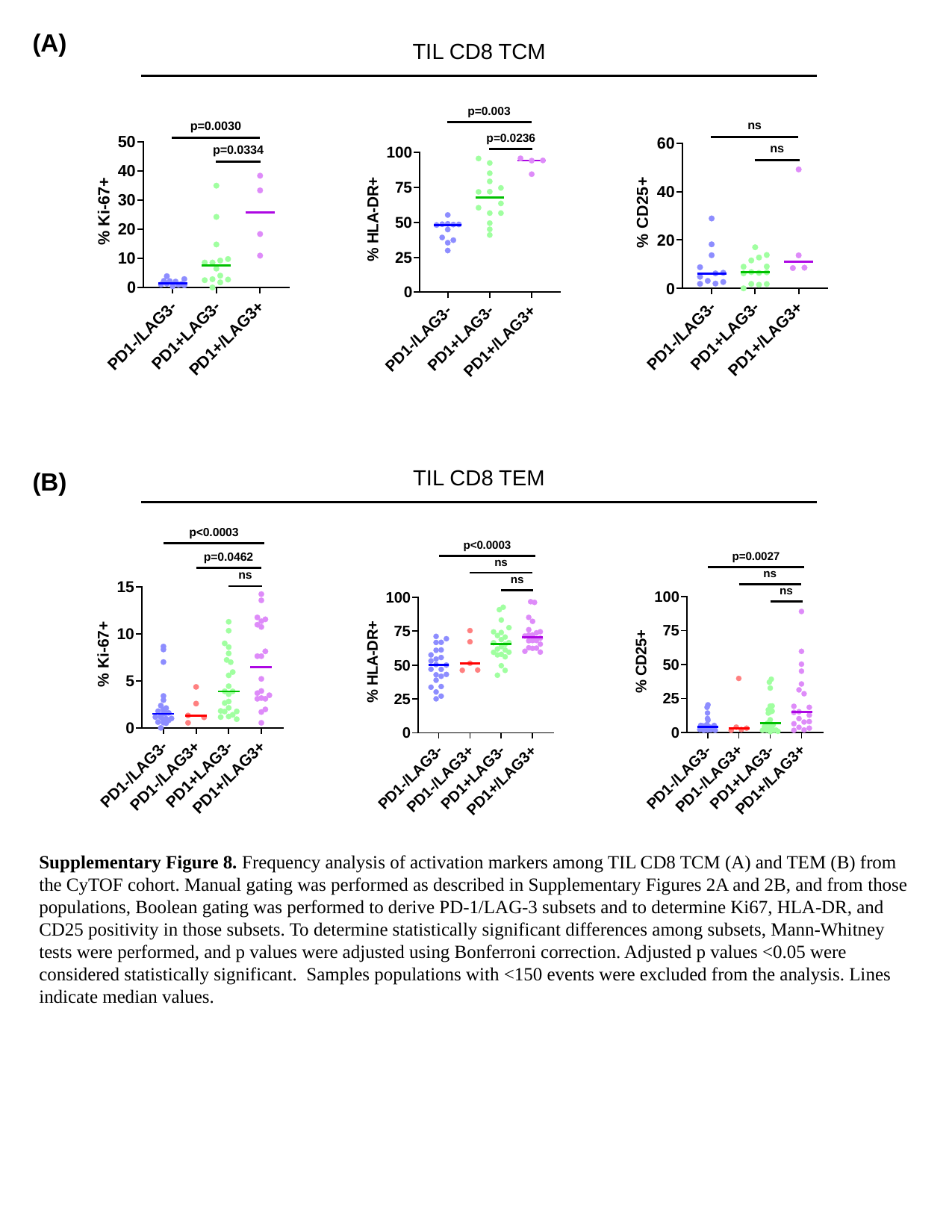

(A)
TIL CD8 TCM
TIL CD8 TEM
(B)
Supplementary Figure 8. Frequency analysis of activation markers among TIL CD8 TCM (A) and TEM (B) from the CyTOF cohort. Manual gating was performed as described in Supplementary Figures 2A and 2B, and from those populations, Boolean gating was performed to derive PD-1/LAG-3 subsets and to determine Ki67, HLA-DR, and CD25 positivity in those subsets. To determine statistically significant differences among subsets, Mann-Whitney tests were performed, and p values were adjusted using Bonferroni correction. Adjusted p values <0.05 were considered statistically significant. Samples populations with <150 events were excluded from the analysis. Lines indicate median values.

## Slide 15
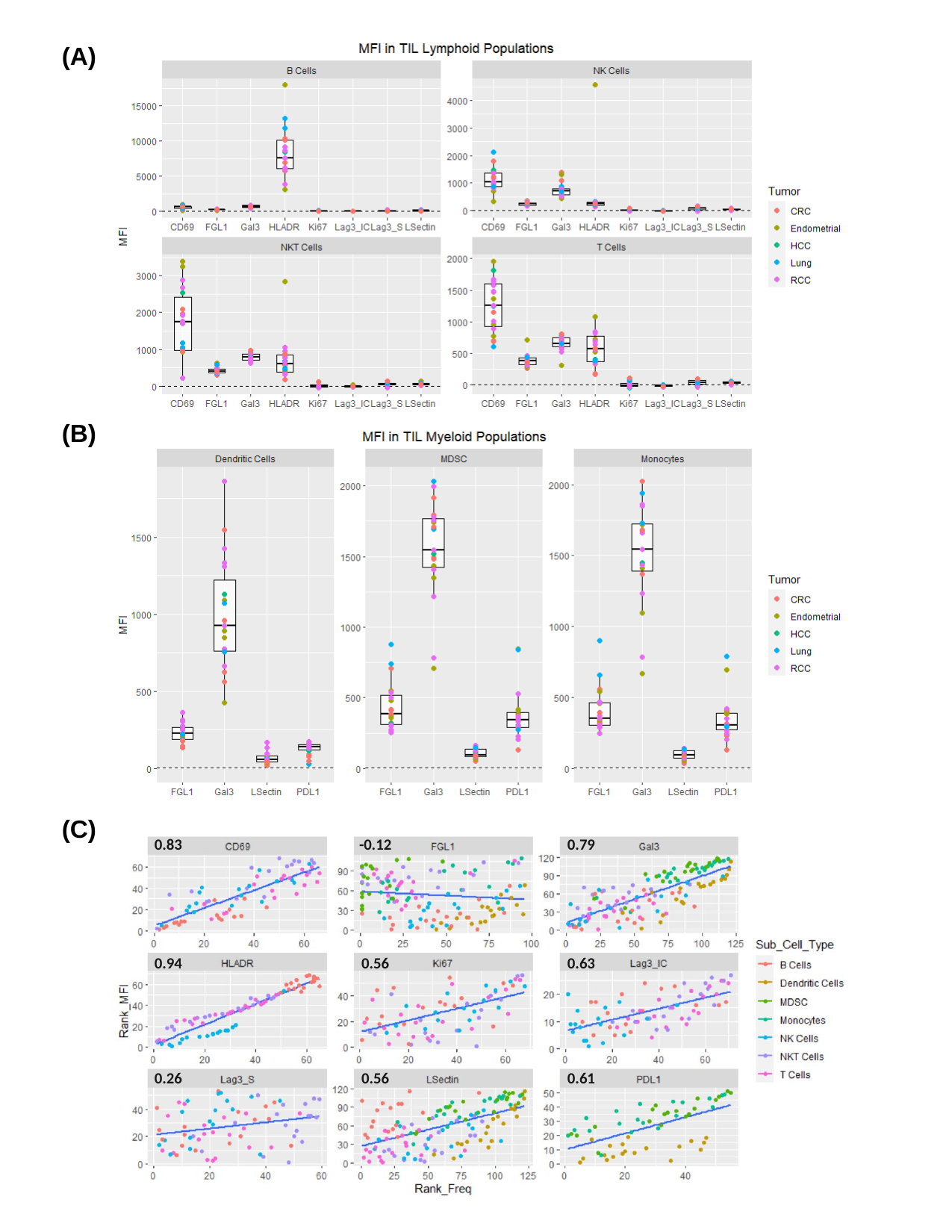

(A)
(B)
(C)
0.83
-0.12
0.79
0.94
0.56
0.63
0.26
0.56
0.61

## Slide 16
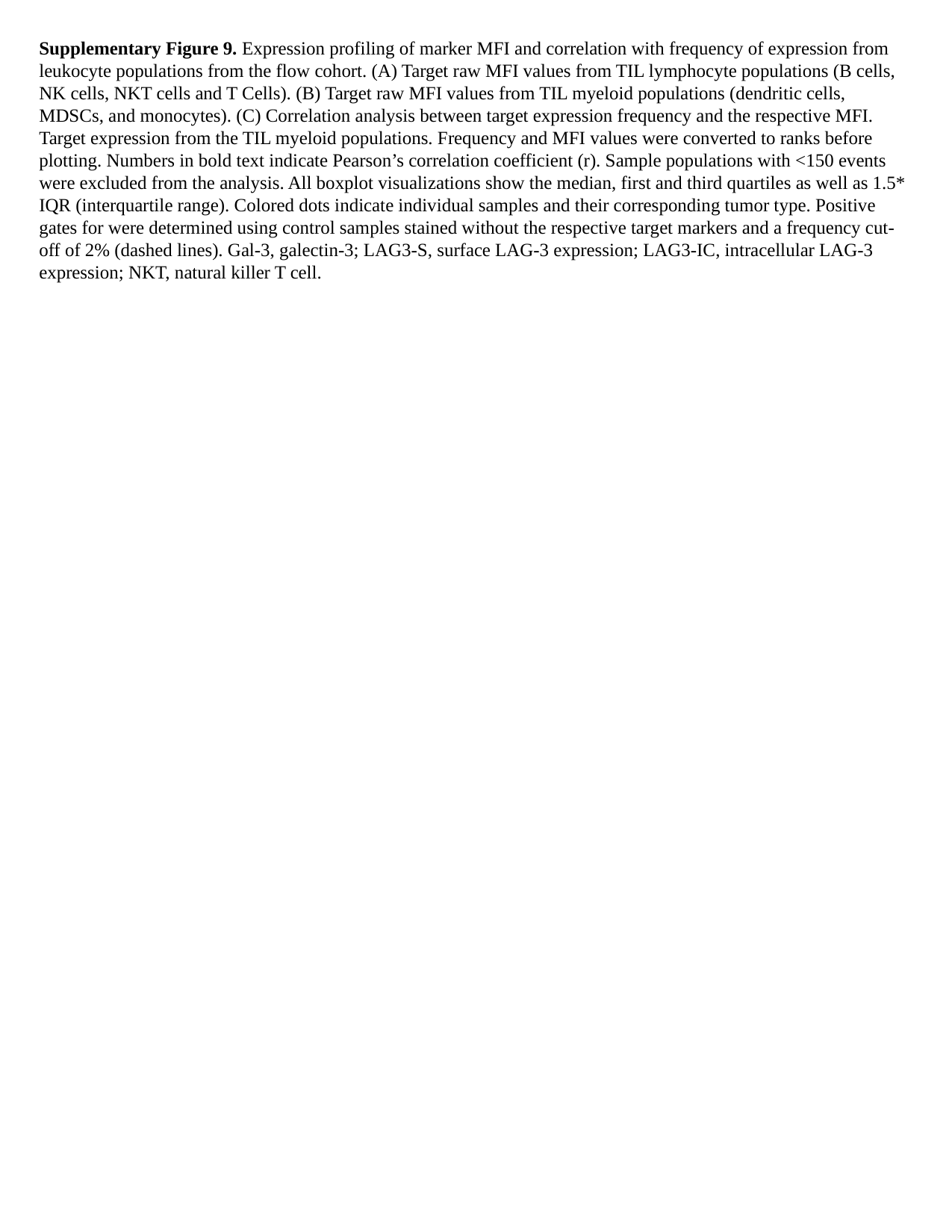

Supplementary Figure 9. Expression profiling of marker MFI and correlation with frequency of expression from leukocyte populations from the flow cohort. (A) Target raw MFI values from TIL lymphocyte populations (B cells, NK cells, NKT cells and T Cells). (B) Target raw MFI values from TIL myeloid populations (dendritic cells, MDSCs, and monocytes). (C) Correlation analysis between target expression frequency and the respective MFI. Target expression from the TIL myeloid populations. Frequency and MFI values were converted to ranks before plotting. Numbers in bold text indicate Pearson’s correlation coefficient (r). Sample populations with <150 events were excluded from the analysis. All boxplot visualizations show the median, first and third quartiles as well as 1.5* IQR (interquartile range). Colored dots indicate individual samples and their corresponding tumor type. Positive gates for were determined using control samples stained without the respective target markers and a frequency cut-off of 2% (dashed lines). Gal-3, galectin-3; LAG3-S, surface LAG-3 expression; LAG3-IC, intracellular LAG-3 expression; NKT, natural killer T cell.
